# Bio-Based Rigid Polyurethane Foams Modified with C-MOF/MWCNTs and TBPBP as Building Insulation Materials: Synergistic Effect and Corresponding Mechanism for Enhancing Fire and Smoke Safety

**DOI:** 10.3390/polym14173630

**Published:** 2022-09-02

**Authors:** Guangxu Bo, Xiaoling Xu, Xiaoke Tian, Jinyong Yan, Xingjian Su, Yunjun Yan

**Affiliations:** Key Laboratory of Molecular Biophysics of the Ministry of Education, College of Life Science and Technology, Huazhong University of Science and Technology, Wuhan 430074, China

**Keywords:** building insulation materials, C-MOF/MWCNTs, phosphorus–nitrogen-containing reactive flame retardant (TBPBP), bio-based rigid polyurethane foam, fire and smoke safety, environmental protection

## Abstract

Rigid polyurethane foams (RPUFs) as building insulation materials quickly burn and release a lot of heat, smoke, and carbon monoxide, and cause human safety risk and severe environmental pollution. To mitigate these disadvantages, MOF/MWCNTs were fabricated via mixing Cu ions’ partly substituted framework of ZIF-67 and MWCNTs, and further calcinated MOF/MWCNTs (C-MOF/MWCTs) was newly generated by calcinating MOF/MWCNTs in air. Then, MOF/MWCNTs and C-MOF/MWCNTs were respectively employed together with a phosphorus–nitrogen-containing reactive flame retardant (TBPBP) to prepare renewable bio-based rigid polyurethane foam, including RPUF-T/MOF/MWCNTs 2 and RPUF-T/C-MOF/MWCNTs 2. The characterization results showed that RPUF-T/C-MOF/MWCNTs 2 had better performance than RPUF-T/MOF/MWCNTs 2 and neat RPUF. Compared to neat RPUF, the compressive strength, limiting oxygen index value, and the mass char residue in cone calorimetry test of RPUF-T/C-MOF/MWCNTs 2, respectively, were increased by 105.93%, 46.35%, and 347.32%; meanwhile, the total heat release rate, total smoke production, total carbon monoxide product, and total carbon dioxide product were reduced by 47.97%, 50.46%, 41.38%, 43.37%, respectively. This study provides a referable method for preparing RPUFs with good physical properties, fire, and smoke safety, which is favorable for human safety and environmental protection as new building insulation materials.

## 1. Introduction

Bio-based polyurethanes (PUs) mostly derived from renewable bio-based polyols or isocyanates are beneficial to their development in view of environmental protection and sustainability issues [1]. Among bio-based PUs, bio-based rigid polyurethane foam (RPUFs) accounts for a large proportion because they possess high specific strength, low heat conductivity, light weight, and low cost, resulting in their widespread use in various fields as thermal insulation materials [2,3]. Nonetheless, bio-based RPUFs burn easily and release a lot of heat, smoke, and carbon monoxide during their combustion. In order to protect human property and life safety and our environment, it is critical to endow bio-based RPUFs with flame-retardant and smoke-suppressing properties.

Until now, review papers aimed at enhancing the flame retardancy and reducing the smoke emission of bio-based RPUFs have been reported [1,4,5]. Many flame retardants are covered in these articles, such as montmorillonite, expandable graphite, carbon tubes, tetrabromophthalic anhydride, (3-aminopropyl)triethoxysilane, 9,10-dihydro-9-oxa-10-phosphaphenanthrene-10-oxide, tri(epoxized-eugenyl)phosphate, and so on. Nevertheless, there are some deficiencies of bio-based RPUFs that could be further improved, such as edible vegetable oil, relatively low compressive performance, somewhat complex preparation process, low flame retardancy, high peak heat release rate (pHRR), etc.

Metal organic frameworks (MOFs) consisting of organic ligands and metal ions have the advantages of good crystallization, flexible chain structure, and high specific surface area, which recently have attracted a lot of interest in many fields, such as catalysis, drug delivery, energy, and separation [6,7]. MOFs can also transform into various porous carbons or porous metal oxides reducing the fire and smoke risk of polymers [8]. Xu et al. reported functionalized reduced graphene oxide (RGO) with Co-ZIF (zeolitic imidazolate frameworks-67) adsorbed borate ions (ZIF-67/RGO-B), which was prepared to reduce the fire risk of epoxy resin [6]. Stephan et al. reported that a metal-organic polymer [Cu (5-methylisophtanalate)] gained stable activity in the oxidation of CO to CO_2_ at 202 °C or higher, which is better than that of Li-containing MOF [9]. Xu et al. prepared the core-shell structure (ZIF-8@MA) via melamine (MA)-coated zeolitic imidazolate framework-8 (ZIF-8), and the ternary composite ZMD was synthesized with the diatomite modified ZIF-8@MA [10]. The study found that the use of ZMD enhanced the flame retardancy and smoke suppression of RPUF.

Combined with studies by other researchers, MOFs were used together with other flame retardants to reduce total heat release (THR), total smoke production (TSP), peak smoke production rate (pSPR), and peak CO production (pCOP), etc. Considering the advantages that multi-walled carbon nanotubes (MWCNTs) could enhance the mechanical properties and thermal stability and promote the formation of stable carbon residues for polymers, two kinds of MOFs and MWCNTs composite materials were prepared. MOF/MWCNTs were fabricated via mixing Cu ions partly substituted framework of ZIF-67 and MWCNTs, and C-MOF/MWCNTs were newly gained by calcinating MOF/MWCNTs in air. MOF/MWCNTs and C-MOF/MWCNTs were respectively added into bio-based RPUFs for studying the effect on improving the physical properties and the flame retardancy and reducing THR, TSP, pSPR, pCOP, etc. MOF/MWCNTs and C-MOF/MWCNTs were also used together with a phosphorus-nitrogen-containing reactive flame retardant (denoted as TBPBP in our previous study [11]) to respectively prepare renewable bio-based RPUFs, further researching the improvement of physical properties as well as environmentally friendly flame-retardant and smoke-suppressing properties. The study found that among many RPUFs, RPUF prepared by compounding C-MOF/MWCNTs and TBPBP achieved the best comprehensive performance.

## 2. Materials and Methods

### 2.1. Materials

Multi-walled carbon nanotubes (MWCNTs) (>97%, tube length = 15–30 um, tube diameter = 3–15 nm) were purchased from Shenzhen Turin Evolution Technology Co., Ltd. (Shenzhen, China). Polyvinylpyrrolidone (PVP) K30, absolute methanol, copper(II) nitrate trihydrate (Cu(NO_3)2_·3H_2_O), cobalt(II) nitrate hexahydrate (Co(NO_3_)_2_·6H_2_O), poly-ethylene glycol-200 (PEG-200), tris(dimethyla-minomethyl)phenol (DMP-30), were bought from China National Pharmaceutical Group Co., Ltd. (Beijing, China). 2-Methylimidazole (2-MIM) (98%) was commercially obtained from Shanghai Aladdin Biochemical Technology Co., Ltd. (Shanghai, China). Modified castor oil polyol (MCOP) (hydroxyl value = 296 mgKOH/g, viscosity = 575 mPas) was bought from Shanghai Joule New Material Tech. Co., Ltd. (Shanghai, China). Polymeric diphenylmethane diisocyanate (pMDI) 44v20 (NCO% = 30.0–32.0) and silicone oil (all T.P. grade) were purchased from Chengdu Advanced Polymer Tech. Co., Ltd. (Chengdu, China). Deionized water and Tetraethyl (1,5-bis(bis(2-hydroxypropyl)amino)pentane-1,5-diyl)bis(phosphonate) (TBPBP) were generated in our laboratory.

### 2.2. Preparation of MOF/MWCNTs and C-MOF/MWCNTs

**Scheme 1:**
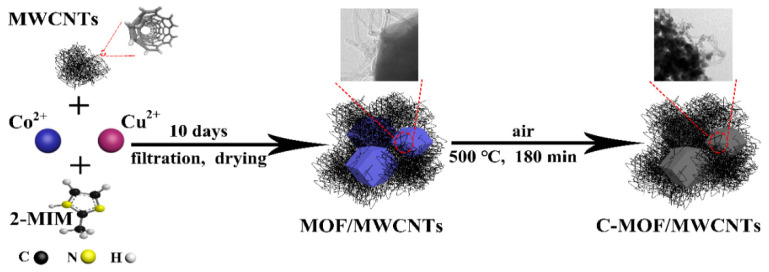
The synthetic route of MOF/MWCTs and C-MOF/MWCNTs.

In total, 1 g MWCNTs, 3 g PVP K30, and 500 mL absolute methanol were added into a 1000 mL glass beaker, then the glass beaker was placed in an ultrasonic cleaner with continuous ultrasonic vibration for 60 min, which made MWCNTs and PVP K30 evenly dispersed in absolute methanol. Then, 4.832 g Cu(NO_3_)_2_·3H_2_O and 11.640 g Co(NO_3_)_2_·6H_2_O were doped into the above beaker with continuous ultrasonic vibration for 60 min. Next, 49.284 g 2-MIM was dispersed into 200 mL absolute methanol in a 500 mL glass beaker. Then, the solution in the 500 mL beaker was poured into the 1000 mL beaker above. The 1000 mL beaker was placed on magnetic stirrer and stirred at a rate of 400 rpm for 20 min. The 1000 mL beaker loaded with the above reagents was placed in a stable place and left to stand for 10 days. Blue-purple metal organic frameworks mixing with multi-walled carbon nanotubes (MOF/MWCTs) were obtained by filtration and drying. Absolute methanol was again gained via rotating evaporation and reused in the next preparation of MOF/MWCTs. Prepared MOF/MWCTs were transferred into a muffle furnace so as to calcine at 500 °C for 180 min in air. Black calcined MOF/MWCTs (denoted as C-MOF/MWCTs) were obtained. The synthetic route of MOF/MWCTs and C-MOF/MWCTs were presented in Scheme 1.

### 2.3. Preparation of Bio-Based Rigid Polyurethane Foams (RPUFs)

**Scheme 2:**
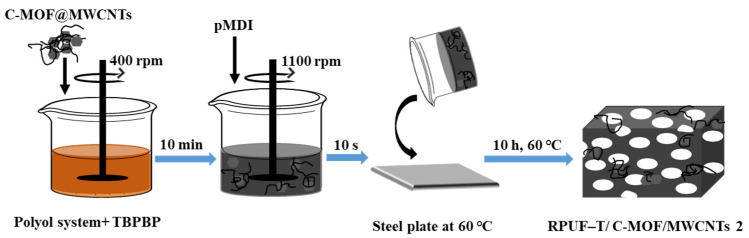
The preparation diagram of bio-based RPUF-T/C-MOF/MWCNTs 2.

Apart from pMDI, the remaining reagents in Table 1 were added to a plastic beaker and stirred at the rate of 400 rpm for 10 min. Subsequently, the corresponding mass of pMDI was swiftly poured into the plastic beaker when the stirring rate was 1100 rpm. After about 10 s, the components in the beaker were rapidly poured onto a steel plate at 60 °C for free foaming. After ca. 3 min, the steel plate was transferred to a vacuum drying oven and dried at 60 °C for 10 h. Bio-based RPUFs were fabricated and then cut down to the appropriate size for various tests. The synthesis diagram of RPUF-T/C-MOF/MWCNTs 2 was presented in Scheme 2. Other RPUFs were prepared via utilizing similar preparation processes to that of RPUF-T/C-MOF/MWCNTs 2.

### 2.4. Characterization

Field emission scanning electron microscope (FSME) images and energy dispersive X-ray spectroscopy (EDXS) images of MOF/MWCTs and C-MOF/MWCTs, respectively, were gained by a field emission scanning electron microscope (ZEISS sigma500, Carl Zeiss AG, Oberkohen, Germany) with energy dispersive X-ray spectrometer (Bruker XFlash 6130, Bruker Corporation, Karlsruher, Germany) at the voltage of 10 kV.

FSME images of the microstructures of the section and char residues of RPUFs were attained via field emission scanning electron microscopes (Sirion 200, FEI Company, Hillsboro, OR, USA) and (EM-30 Plus, COXEM Co., Ltd., Daejeon City, Korea).

Transmission electron microscopy (TEM) images and energy dispersive X-ray spectroscopy (EDXS) images of MOF/MWCTs, C-MOF/MWCTs, and char residues of RPUFs, respectively, were obtained via a transmission electron microscope (JEOL-JEM 2100 F, JEOL, Showa, Tokyo, Japan) with energy dispersive X-ray spectrometer at the voltage of 80 kV. Before the TEM test, MOF/MWCTs and C-MOF/MWCTs were respectively added into alcohol, and then the mixture was ultrasonic in an ultrasonic cleaning machine.

X-ray diffraction (XRD) data of MOF/MWCTs and C-MOF/MWCTs were garnered on a diffractometer Empyrean (PANalytical B.V., Panakot, The Netherlands) with Cu Kα radiation (λ = 0.154 nm) at 30 mA and 40 kV in the range 2θ = 5–90° lasting 5 min.

The testing methods of Fourier-transform infrared (FT-IR), X-ray photoelectron spec-troscopy (XPS), thermal conductivity, density, compressive strength, thermal gravimetric analysis (TGA), limiting oxygen index (LOI), vertical burning, Laser Raman spectroscopy, and cone calorimeter (CC) of MOF/MWCTs, C-MOF/MWCTs, RPUFs, and char residues of RPUFs refer to the article.

## 3. Results and Discussion

### 3.1. Characterization of C-MOF/MWCNTs

**Figure 1:**
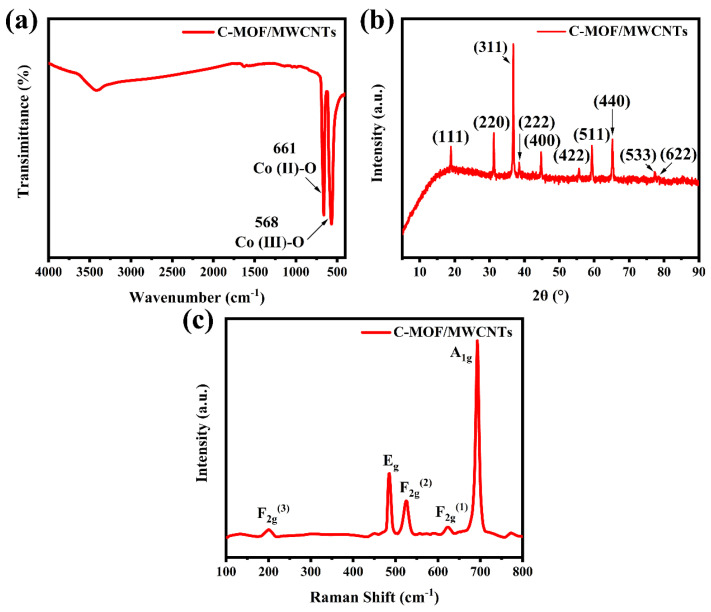
FT-IR spectrum (**a**), XRD curve (**b**), Raman curve (**c**) of C-MOF/MWCNTs.

Physical and chemical properties of C-MOF/MWCNTs were tested before it was incorporated into RPUFS. Fourier-transform infrared (FT-IR) spectrum, X-ray diffraction (XRD) curve, and Raman curve of C-MOF/MWCNTs were exhibited in Figure 1a–c, respectively. Figure 1a shows two very strong characteristic peaks at 568 cm^−1^ and 661 cm^−1^, which were respectively ascribed to the stretching vibration of Co(III)-O and Co(II)-O of Co_3_O_4_ [12,13] that was a calcined product of MOF in air. As can be seen from Figure 1b, some diffraction peaks at 19.05°, 31.24°, 36.76°, 38.51°, 44.82°, 55.66°, 59.30°, 65.23°, 77.33°, and 78.40° belonged to the plane diffraction patterns ((111), (220), (311), (222), (400), (422), (511), (440), (533), (622)) of Co_3_O_4_ [6,14]. As shown in Figure 1c, the five typical peaks at around 201 cm^−1^, 485 cm^−1^, 524 cm^−1^, 622 cm^−1^, and 693 cm^−1^ were consistent with F_2g_^(3)^, Eg, F_2g_^(2)^, F_2g_^(1)^, and A_1g_ modes of Co_3_O_4_ [15]. In fact, CuO was theoretically created via calcining MOF in air. Interestingly, no obvious characteristic peaks of CuO were observed in Figure 1a–c, which were presumably attributed to low fraction of CuO and amorphous CuO.

**Figure 2:**
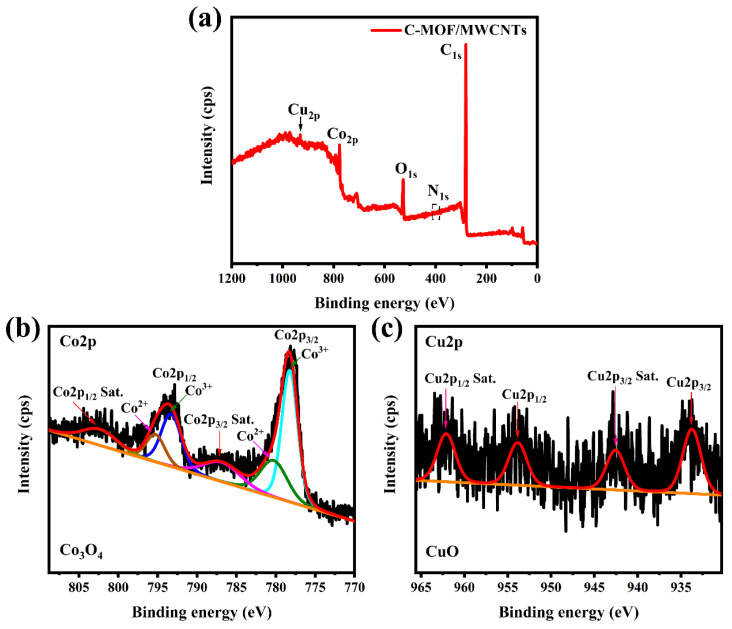
XPS curves of C-MOF/MWCNTs (**a**), its Co2p (**b**), and Cu2p (**c**).

X-ray photoelectron spectroscopy (XPS) curves of C-MOF/MWCNTs, its Co2p and Cu2p were present in Figure 2a–c, respectively. Compared with XPS curves of MOF/MWCNTs (see Figure S2 in Supplementary Materials [16,17,18,19,20,21,22,23,24]), the peak of N1s vanished in Figure 2a, which was assigned to the thermal decomposition of 2-MIM of MOF/MWCNTs in the period of calcination in air. The proportion of specific elements of MOF/MWCNTs and C-MOF/MWCNTs were shown in Table S3. As shown in Table S3, the proportion of C and N atoms decreased, and the proportion of O, Co, and Cu atoms increased. The proportion of C atoms did not decrease significantly, and still accounted for 70% of the atomic proportion, which was caused by the large amount of MWCNTs contained in C-MOF/MWCNTs, because the mass loss of MWCNTs almost did not occur when calcined in air atmosphere of 500 °C. The reason for the increase in the proportion of O atoms was that MOF reacted with oxygen to form CO_3_O_4_ and CuO during the calcination in air atmosphere. The XPS spectrum of Co2p showed two characteristic peaks and two satellite peaks [25,26]. The peak of Co2p_3/2_ was divided into two peaks with the binding energies of 778.5 eV and 780.6 eV for Co^3+^2p_3/2_ and Co^2+^2p_3/2_ species, respectively. The peak of Co2p1/2 was divided into two peaks at around 793.4 eV and 795.5 eV, respectively, which belong to Co^3+^2p_1/2_ and Co^2+^2p_1/2_ species. The results of Figure 2b displayed the coexistence of Co^3+^ and Co^2+^ in C-MOF/MWCNTs. The Cu2p XPS spectrum (Figure 2c) exhibited four peaks at near 933.8 eV, 942.6 eV, 953.8 eV, and 953.6 eV, which were assigned to Cu2p_3/2_, the satellite peak of Cu2p_3/2_, Cu2p_1/2_, and the satellite peak of Cu2p_1/2_ [15]. Evidently, compared with the Cu2p XPS spectrum of MOF/MWCNTs (Figure S2c), the Cu2p XPS spectrum of C-MOF/MWCNTs fit more precisely because of the increased atomic ratio of Cu via the calcination of MOF/MWCNTs.

**Figure 3:**
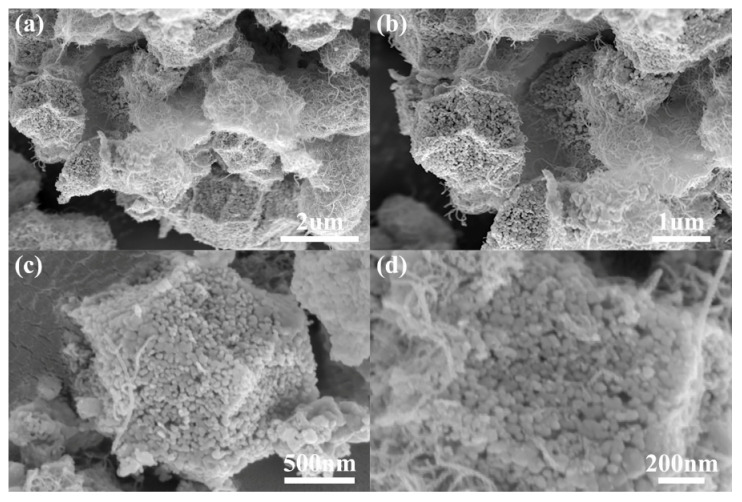
FSEM images of C-MOF/MWCNTs at various magnifications. (**a**) under the field of view of 2 um; (**b**): under the field of view of 1 um; (**c**): under the field of view of 500 nm; (**d**): under the field of view of 200 nm.

The microstructure of C-MOF/MWCNTs was detected via field emission scanning electron microscope (FSME), and the corresponding images at various magnifications were presented in Figure 3. Compared with FSEM images of MOF/MWCNTs (Figure S3), C-MOF/MWCNTs maintained the fundamental frame structure of MOF/MWCNTs. In detail, MWCNTs still existed after calcination, and calcined MOF retained the normal dodecahedron structure, whereas the surface of MOF altered from firm to porous because of the decomposition of the organic linker during calcination [26]. FSEM image of C-MOF/MWCNTs with mapping mode and its specific energy dispersive X-ray spectroscopy (EDXS) elemental mapping were shown in Figure S12. The mass ratio and atom ratio of elements of FSEM image of C-MOF/MWCNTs with mapping mode were exhibited in Table S4. Figure S12 and Table S4 illustrate that C-MOF/MWCNTs were mainly composed of C, O, Co elements, and also contained a very small amount of Cu element, and basically did not contain N element. The result was consistent with that of XPS, but the specific content of the elements was different. This may be due to the different accuracy of the test equipment or different sampling sites.

**Figure 4:**
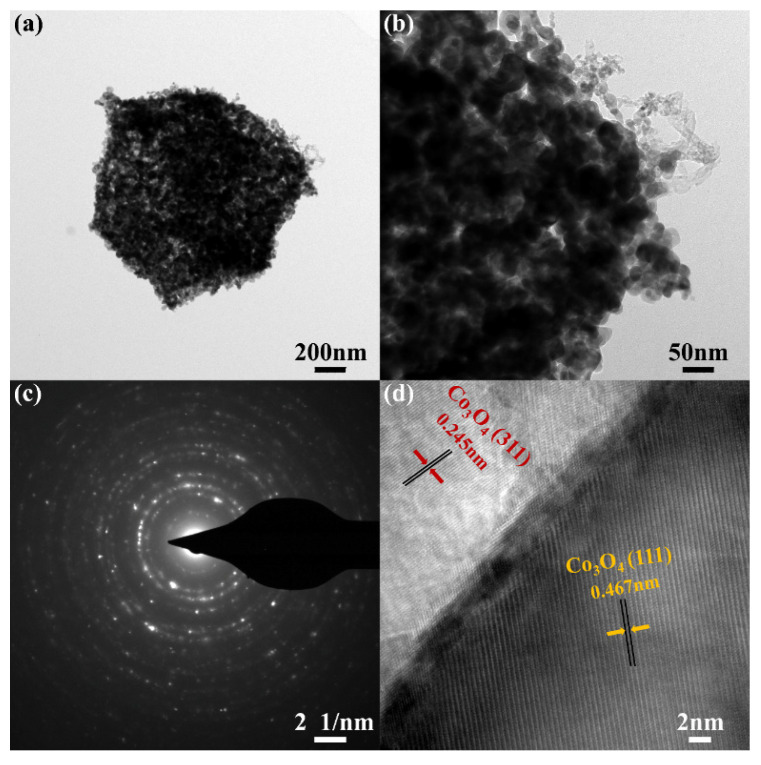
TEM images of C-MOF (**a**,**b**), SAED pattern of C-MOF (**c**), HRTEM image of C-MOF (**d**).

Transmission electron microscopy (TEM) images, selected area electron diffraction (SAED) pattern and high-resolution TEM (HRTEM) image of C-MOF were presented in Figure 4. From Figure 4a,b, the surface of C-MOF possessed many holes and was wrapped in MWCNTs, which was in accordance with the FSEM results of C-MOF. Figure 4c shows that SAED pattern of C-MOF revealed that it had the polycrystalline properties. In addition, the lattice planes of Co_3_O_4_ ((111), (311)) were observed in the HRTEM image of C-MOF (Figure 4d). Unfortunately, the lattice planes of CuO were detected via the SAED pattern, HRTEM, and XRD, which were presumably attributed to a low fraction of CuO or amorphous CuO as mentioned above. TEM image of C-MOF with mapping mode (Figure S13a), EDXS elemental mapping (Figure S13b–f), EDXS curve (Figure S13g) of C-MOF, and the mass ratio and atom ratio of elements of TEM image of C-MOF with mapping mode (Table S5) also proved, although Cu element’s atomic percentage increased slightly, but it was still low.

The above characterization revealed that with the decomposition of organic ligands, MOF were converted to major Co_3_O_4_ and minor CuO during the calcination at 500 °C for 3 h in air atmosphere.

**Figure 5:**
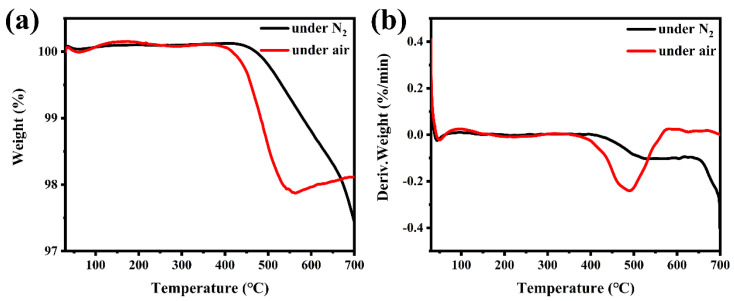
TGA curves of C-MOF/MWCNTs under N_2_ and air (**a**), and DTG curves under N_2_ and air (**b**).

Thermo gravimetric analyzer (TGA) curves and DTG (derivative thermogravimetry) curves under N_2_ and air, DTG curve under N_2_ and air of C-MOF/MWCNTs, respectively, were presented in Figure 5a,b. From Figure 5a,b, there was almost no mass loss for C-MOF/MWCNTs either under N_2_ or air, because the organic ligands had almost been decomposed during the calcination to prepare C-MOF/MWCNTs. It was worth mentioning that the mass of C-MOF/MWCNTs started to increase slowly after 550 °C under air because of the introduction of oxygen into the residue. Apparently, C-MOF/MWCNTs had better thermal stability compared to MOF/MWCNTs.

### 3.2. Characterization of RPUFs

**Figure 6:**
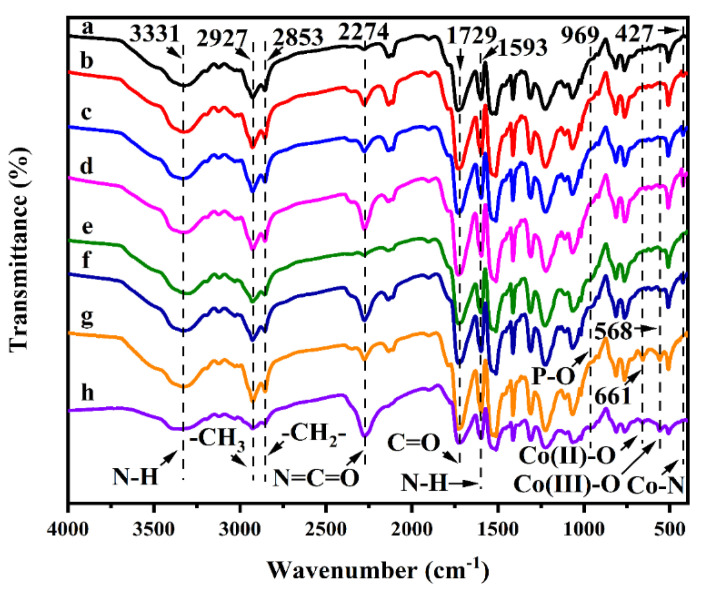
FT-IR spectra of all RPUFs (**a**): neat RPUF, (**b**): RPUF-MOF/MWCNT 1, (**c**): RPUF-MOF/MWCNT 2, (**d**): RPUF-MOF/MWCNT 3, (**e**): RPUF-T, (**f**): RPUF-T-MOF/MWCNT 2, (**g**): RPUF-C-MOF/MWCNT 2, (**h**): RPUF-T/C-MOF/MWCNT 2.

**Figure 7:**
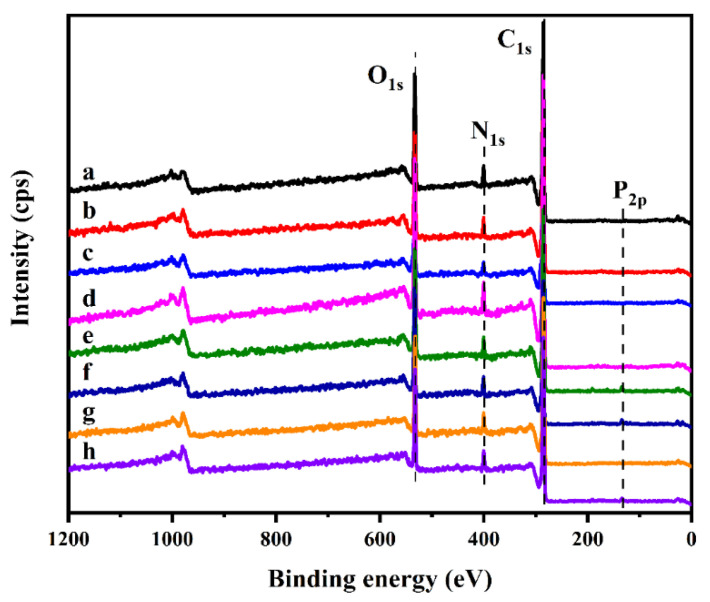
XPS curves of all RPUFs (**a**): neat RPUF, (**b**): RPUF-MOF/MWCNT 1, (**c**): RPUF-MOF/MWCNT 2, (**d**): RPUF-MOF/MWCNT 3, (**e**): RPUF-T, (f): RPUF-T-MOF/MWCNT 2, (**g**): RPUF-C-MOF/MWCNT 2, (**h**): RPUF-T/C-MOF/MWCNT 2.

RPUFs’ chemical component has a tremendous influence on their various properties, such as mechanical properties, flame retardant properties, etc. FT-IR and XPS were carried out identifying the chemical component and structure of RPUFs, and the corresponding testing results were shown in Figure 6 and Figure 7, respectively. As presented in Figure 6, the peaks of all RPUFs at 3331 cm^−1^, 2927 cm^−1^, 2853 cm^−1^, and 1729 cm^−1^ were respectively assigned to the stretching vibrations of N-H, -CH_3_, -CH_2_-, and C=O, and the peaks at 2274 cm^−1^ were ascribed to N=C=O anti-symmetric stretching vibrations, and the peaks of N-H bending vibrations were detected at 1593 cm^−1^ for all RPUFs, and the peaks at 969 cm^−1^ for P-O stretching vibrations was tested in the FT-IR curves of RPUF-T, RPUF-T/MOF/MWCNTs 2 and RPUF-T/C-MOF/MWCNTs 2 on account of the insertion of TBPBP into the copolyester chains [11,27]. Peaks at 424 cm^−1^ for RPUF-MOF/MWCNTs 1, RPUF-MOF/MWCNTs 2, RPUF-MOF/MWCNTs 3, and RPUF-T/MOF/MWCNTs 2 were the stretching vibrations of the Co-N bond because of the use of MOF/MWCTNs. FT-IR spectra of RPUF-MOF/C-MWCNTs 2 and RPUF-T/MOF/C-MWCNTs 2 were all detected in two peaks at 568 cm^−1^ and 661 cm^−1^, which were respectively allocated to the stretching vibration of Co(III)-O and Co(II)-O due to the employment of C-MOF/MWCTNs.

The FT-IR results prove that all RPUFs were successfully prepared, while the peaks of Co and Cu elements were not obviously detected in XPS curves of all RPUFs (Figure 7). The weak peaks of P2p were only found in RPUF-T and RPUF-T/MOF/MWCNTs 2 and RPUF-T/C-MOF/MWCNTs 2, because TBPBP reacted with pMDI and formed the foam matrix, which was likely to be easier to detect.

**Figure 8:**
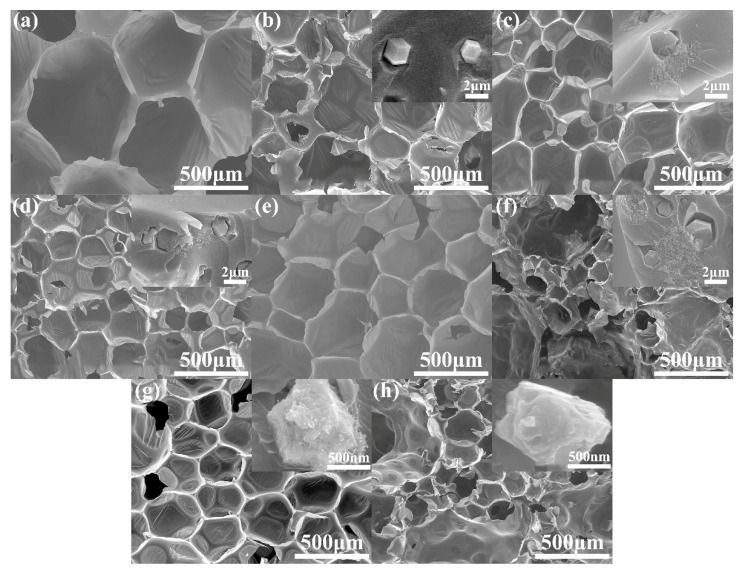
FSEM images of neat RPUF (**a**), RPUF-MOF/MWCNTs 1 (**b**), RPUF-MOF/MWCNTs 2 (**c**), RPUF-MOF/MWCNTs 3 (**d**), RPUF-T (**e**), RPUF- T/MOF/MWCNTs 2 (**f**), RPUF-C-MOF/MWCNTs 2 (**g**), and RPUF-T/C-MOF/MWCNTs 2 (**h**).

The physical and mechanical properties of RPUFs are profoundly influenced by their cell morphology [28,29]. The cell morphology of all RPUFs were observed via FSEM images (Figure 8). The particle size of MOF and C-MOF were mostly distributed in ca. 0.1–2 um via the above FSEM and TEM characterization. The cell size of neat RPUF was about 300–700 um. As the contents of MOF/MWCNTs increased, the cell number became more and the cell size became smaller, because MOF/MWCNTs were as nucleation agents for the bubble’s formation. In particular, the cell size of RPUF-MOF/MWCNTs 1, RPUF-MOF/MWCNTs 2 and RPUF-MOF/MWCNTs 3 were approximately 200–600 um, 100–500 um and 100–400 um, respectively. Remarkably, the RPUF-T/MOF/MWCNTs 2 formed by the use of TBPBP and MOF/MWCNTs had a wide cell size distribution (ca. 50–800 um) because of the uneven foaming resulted from the high viscosity of the foamed system. Meanwhile, the walls of RPUFs adding MOF/MWCNTs were embedded with MOF and MWCNTs. The cell size of RPUF-C-MOF/MWCNTs 2 and RPUF-T/C-MOF/MWCNTs 2 were roughly 100–500 um and 50–800 um, respectively. The cell number and size of RPUFs synthesized with C-MOF/MWCNTs were similar to RPUFs synthesized with the same amount of MOF/MWCNTs.

The mechanical and physical properties considerably restrict the application of RPUFs [30,31]. RPUFs’ mechanical and physical properties generally include density, compressive strength, and thermal conduction coefficient, and the corresponding testing results are summarized in Table 2. According to the test results in Table 2, RPUFs employed MOF/MWCNTs, C-MOF/MWCNTs and TBPBP gained a gradual increase in density, which was considered to be the increase in viscosity of the polyol system [32]. Fortunately, even the largest density (40.9 kg/m^3^) of RPUF-T/MOF/MWCNTs 2 was not much greater than the smallest density (36.7 kg/m^3^) of neat RPUF, which had no influence on the actual application.

Compressive strength is an important parameter, which affected the application of RPUFs [33]. Compressive strength of RPUFs is correlated with many factors, such as the formula, foam density, the cellular size, number and its uniformity, and cell wall thickness [34,35,36]. In general, RPUFs with larger density attain larger compressive strength. The compressive strength of neat RPUF was 236 ± 29 kPa, while the compressive strengths of RPUF-MOF/MWCNTs 1, RPUF-MOF/MWCNTs 2, RPUF-MOF/MWCNTs 3, RPUF-T, RPUF-T/MOF/MWCNTs 2, RPUF-C-MOF/MWCNTs 2, and RPUF-T/C-MOF/MWCNTs were increased to 301 ± 26 kPa, 358 ± 17 kPa, 379 ± 28 kPa, 422 ± 30 kPa, 453 ± 39 kPa, 367 ± 23 kPa, and 486 ± 25 kPa, respectively, which had an increment of 27.54%, 51.69%, 60.59%, 78.81%, 91.95%, 55.51%, and 105.93%, respectively. The compressive strength was greatly upgraded via using MOF/MWCNTs, C-MOF/MWCNTs, and TBPBP, especially when MOF/MWCNTs and TBPBP, or C-MOF/MWCNTs and TBPBP were used together.

Thermal conduction coefficient is a key index of thermal insulation materials [37]. On the whole, the thermal conduction coefficient of RPUFs was relatively low. Even though the maximum thermal conduction coefficient of RPUF-T/MOF/MWCNTs 2 was 35.53 × 10^−3^ W/(mK), which was not much larger than 31.67 × 10^−3^ W/(mK) of neat RPUF. The thermal conduction coefficient of RPUFs (λ_R_) was calculated by the equation: λ_R_ = λ_s_ + λ_g_ + λ_c_ + λ_r_, (λ_s_ = thermal conductivity of the solid phase, λ_g_ = thermal conductivity of the gas in the cells, λ_c_ = thermal conductivity attributed to convection within cells, λ_r_ = thermal conductivity assigned to radiation across the cells) [38,39]. As far as the thermal conduction coefficient of other foams was higher than that of neat RPUF for two reasons. On the one hand, the increase in density increased the thermal conduction coefficient for RPUFs, which was assigned to the progressively accrescent contribution of solid thermal conductivity [40]. Additionally, the contribution of solid heat conduction to thermal conduction coefficient is considerably larger than that of thermal convection and thermal radiation [40]. On the other hand, cell size, cell number, and cell wall thickness affected the thermal conduction coefficient of RPUFs via influencing photon transfers, because the thermal conduction coefficient (0.025 mW/(mK) of air is higher than for the CO_2_ (0.015 mW/(mK)) [41,42,43].

**Figure 9:**
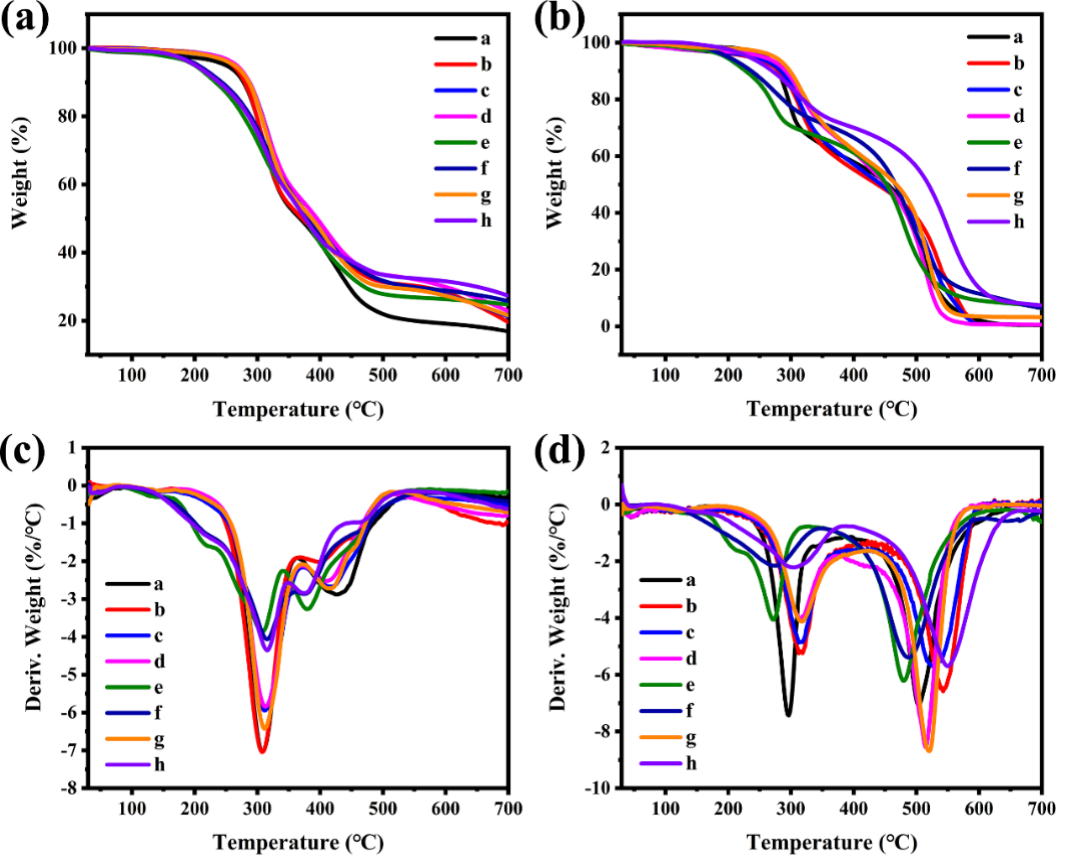
TGA curves of RPUFs under N_2_ (**a**) and under air (**b**), DTG curves of RPUFs under N_2_ (**c**) and under air (**d**). (a: neat RPUF; b: RPUF-MOF/MWCNTs 1; c: RPUF-MOF/MWCNTs 2; d: RPUF-MOF/MWCNTs 3; e: RPUF-T; f: RPUF-T/MOF/MWCNTs 2; g: RPUF-C-MOF/MWCNTs 2; h: RPUF-T/C-MOF/MWCNTs 2).

Thermo gravimetric analyzer (TGA) is commonly used for assessing the thermal stability of polymers [44]. As illustrated in Figure 9, TGA curves and DTG curves of RPUFs were examined under N_2_ and air, respectively. Some important indexes, namely the 5% weight loss temperature (T_5%_), the 50% weight loss temperature (T_50%_), the maximum rate of weight loss (Rmax), the temperature of maximum rate (Tmax), and the residue yield, were exhibited in Table S6 (under N_2_) and Table S7 (under air).

By observing Figure 9, two main degradation processes existed for all RPUFs under both N_2_ and air atmospheres; the first degradation stage was assigned to the dissociation of the low-molecular-weight compounds, as polyurethane hard segments, and the second degradation stage was considered to the dissociation of the high-molecular-weight compounds, as polyurethane soft segments [45,46,47]. It is interesting that the first degradation stage under both N_2_ and air atmospheres had the same pyrolysis mechanism and all happened ca.200–350 °C, while the temperature of the second degradation stage under air (400–600 °C) was higher than that under N_2_ (350–500 °C) and the degradation rate under air accelerated significantly due to the oxidation reaction caused by oxygen [48]. Thus, the residue mass of the same sample under air was much lower than that under N_2_. As for the influences of MOF/MWCNTs or C-MOF/MWCNTs on the thermal degradation of RPUFs under N_2_, T_5%_ and T_50%_ were both increased and Rmax_1_ was decreased; while Rmax_2_ was not changed abruptly, the residue at 700 °C was significantly increased. All in all, the utilization of MOF/MWCNTs or C-MOF/MWCNTs independently improved the thermal stability of RPUFs. Remarkably, when TBPBP and MOF/MWCNTs or TBPBP and C-MOF/MWCNTs were used together, the prepared RPUF-T/MOF/MWCNTs 2 or RPUF-T/C-MOF/MWCNTs 2 obtained more residues in all RPUFs amount (25.79 wt% or 27.39 wt%), revealing that they possessed good thermal stability. T_5%_ of RPUF-T, RPUF-T/MOF/MWCNTs 2, and RPUF-T/C-MOF/MWCNTs 2 were both ca. 200 °C, lower than these of other RPUFs (over 250 °C), because there was the preferential decomposition of TBPBP due to the early decomposition and poor thermal stability of P-O-C and P-C bonds compared to the C-C bond [11,49]. It was precisely because the preferential decomposition of TBPBP and the simultaneous use of MOF/MWCNTs or C-MOF/MWCNTs promoted the formation of expanded and stable char residues; these were the reasons why RPUF-T/MOF/MWCNTs 2 and RPUF-T/C-MOF/MWCNTs 2 obtained the good thermal stability.

The effect of MOF/MWCNTs or C-MOF/MWCNTs on the thermal degradation of RPUFs under air was similar to those under N_2_. In other words, with the increasing amount of MOF/MWCNTs, RPUFs had gradually better thermal stability under air. However, the thermal stability of RPUFs under air was worse than that under N_2_ because of the presence of oxidizing gases [48]. In terms of T_5%_, T_50%_, residue (wt%), and other indicators, RPUF-C-MOF/MWCNTs 2 had better thermal stability than RPUF-MOF/MWCNTs 2. Because the MOF/MWCNTs in RPUF-MOF/MWCNTs 2 continued to decompose during the TGA test, while the C-MOF/MWCNTs in RPUF-C-MOF/MWCNTs 2 was strongly stable at high temperature as mentioned earlier, resulting in the higher thermal stability of RPUF-T/C-MOF/MWCNTs 2 than that of RPUF-T/MOF/MWCNTs 2.

Vertical burning test and limiting oxygen index (LOI) test are simple and effective methods to investigate the flame retardancy of RPUFs [28,50]. The flame retardant performance of the vertical burning test of RPUFs is assessed via UL-94 ranks. UL-94 ranks and LOI values of RPUFs were listed in Table 3. From Table 3, neat RPUF failed to obtain any UL-94 rank on account of the low break-up temperature of urethane bonds and high porous cellular structure that caused its rapid combustion [46,51]. RPUF-MOF/MWCNTs 1, RPUF-MOF/MWCNTs 2, RPUF-MOF/MWCNTs 3, and RPUF-C-MOF/MWCNTs 2 all did not pass any UL-94 ranks and fast burned in air as well as neat RPUF. this was because MOF/MWCNTs or C-MOF/MWCNTs mixed in RPUFs, which were unable to promote forming the rigid, stable, sufficient chars or releasing non-flammable gases prevented the exchange of heat and combustible gases during RPUFs’ combustion. RPUF-T, RPUF-T/MOF/MWCNTs 2 and RPUF-T/C-MOF/MWCNTs 2 all achieved V-0 rank of UL-94 standards, because TBPBP expedited forming the intact, firm phosphorous-nitrogen-containing chars and releasing non-flammable gases containing P or N elements [11].

LOI value is the lowest oxygen content to maintain combustion. It is clear that high LOI values mean good flame retardant performance for RPUFs. As showed in Table 3, LOI values of neat RPUF, RPUF-MOF/MWCNTs 1, RPUF-MOF/MWCNTs 2, RPUF-MOF/MWCNTs 3, and RPUF-C-MOF/MWCNTs 2 were, respectively, 19.2%, 19.6%, 20.1%, 20.4%, and 20.3%, which were all lower than the proportion of oxygen in the air, explaining that they failed to gain any UL-94 ranks. Meanwhile LOI values of RPUF-T, RPUF-T/MOF/MWCNTs 2, and RPUF-T/C-MOF/MWCNTs 2 achieved 25.6%, 27.4%, and 28.1%, respectively, because of the formation of the intact, firm, intumescent phosphorous-nitrogen-containing chars and the release of contained P or N flame retardant inhibitors as motioned above. Compared to RPUF-T, RPUF-T/MOF/MWCNTs 2, and RPUF-T/C-MOF/MWCNTs 2 possessed higher LOI values owing to the more intact, firm, intumescent char layers caused by the synergistic effect of TBPBP and MOF/MWCNTs or C-MOF/MWCNTs. Remarkably, thanks to the synergistic flame retardant effect of MOF/MWCNTs or C-MOF/MWCNTs and TBPBP, their simultaneous utilization had the more visible effect of enhancing the flame retardant performance of RPUF than using MOF/MWCNTs or C-MOF/MWCNTs or TBPBP alone.

The V-0 rank of UL-94 standards and high LOI values mean that materials are extremely difficult to ignite. Hence, RPUF-T, RPUF-T/MOF/MWCNTs 2, and RPUF-T/C-MOF/MWCNTs 2 are extremely beneficial to environmental protection, especially RPUF-T/C-MOF/MWCNTs 2.

**Figure 10:**
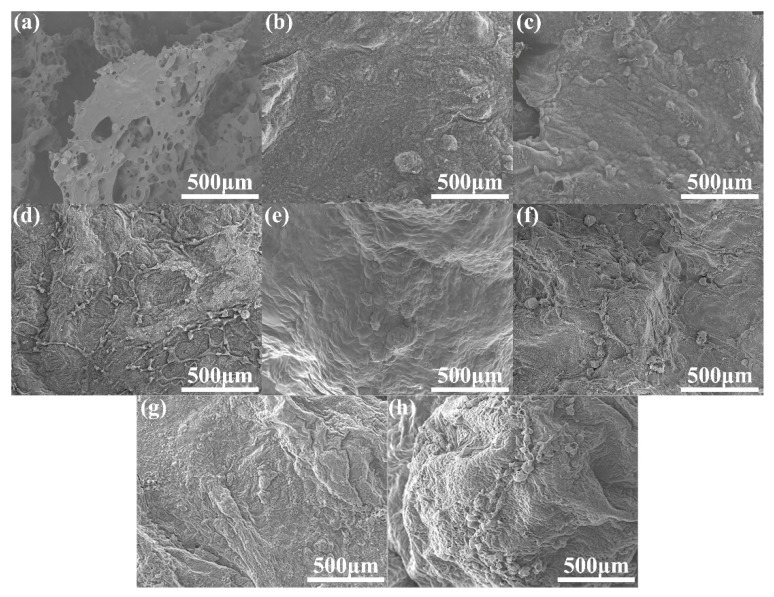
FSEM images of char residues of neat RPUF (**a**), RPUF-MOF/MWCNTs 1 (**b**), RPUF-MOF/MWCNTs 2 (**c**), RPUF-MOF/MWCNTs 3 (**d**), RPUF-T (**e**), RPUF-T/MOF/MWCNTs 2 (**f**), RPUF-C-MOF/MWCNTs 2 (**g**), and RPUF-T/C-MOF/MWCNTs 2 (**h**).

FSEM images of RPUFs’ char residues obtained via ca. 1300 °C flame were presented in Figure 10. The char residue of neat RPUF was loose, holey, and brittle, which could not protect the interior matrix to evade combustion, and the char residues of RPUF-T were in-tumescent and intact, which could protect the interior matrix to avoid the exchange with heat and oxygen and did not play the role of flame retardant performance. The complete, firm char residues of RPUF-MOF/MWCNTs 1, RPUF-MOF/MWCNTs 2, RPUF-MOF/MWCNTs 3, and RPUF-C-MOF/MWCNTs 2 were both found via FSEM on a microscopic scale, but the char residues were formed on a small scale in the later stages of RPUFs combustion and failed to play a good flame retardant effect. The char residues of RPUF-T, RPUF-T/MOF/MWCNTs 2, RPUF-T/C-MOF/MWCNTs 2 were firm, intact, and intumescent, which acted as flame-retardant barriers in the condensed phase, which resulted in more foam matrix turning into char residues instead of flue gas that pollutes the environment.

**Figure 11:**
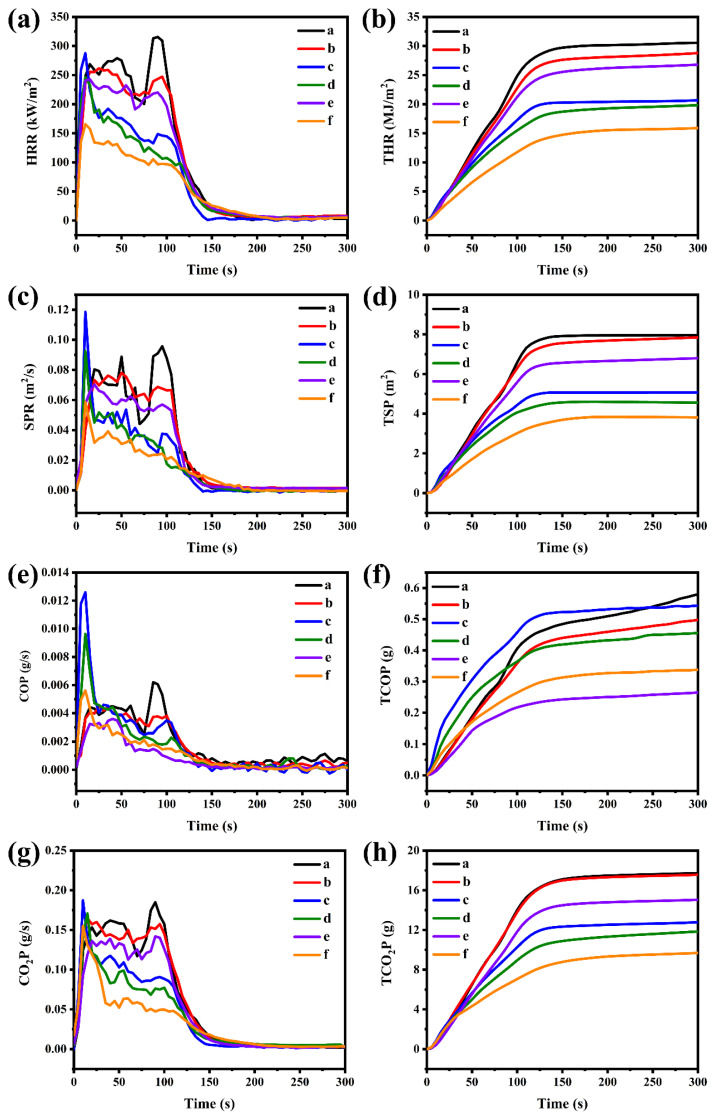
HRR curves (**a**), THR curves (**b**), SPR curves (**c**), TSP curves (**d**), COP curves (**e**), TCOP curves (**f**), CO2P curves (**g**), and TCO2P (**h**) of neat RPUF, RPUF-MOF/MWCNTs 2, RPUF-T, RPUF-T/MOF/MWCNTs 2, RPUF-C-MOF/MWCNTs 2, and RPUF-T/C-MOF/MWCNTs 2.

Cone calorimeter (CC) test can simulate real combustion of RPUFs and acquire sufficient combustion data including the time to ignition (TTI), average effective heat combustion (av-EHC), heat release rate (HRR), total heat release rate (THR), peak heat release rate (pHRR), smoke production rate (SPR), total smoke production (TSP), peak smoke production rate (pSPR), CO production (COP), total COP (TCOP), CO_2_ production (CO_2_P), total CO_2_P (TCO_2_P), and char residues etc. Combined with the above results and analysis, six RPUFs (namely, neat RPUF, RPUF-MOF/MWCNTs 2, RPUF-T, RPUF-T/MOF/MWCNTs 2, RPUF-C-MOF/MWCNTs 2, and RPUF-T/C-MOF/MWCNTs 2) were chosen for CC test. The corresponding curves of HRR, THR, SPR, TSP, COP, TCOP, CO_2_P, and TCO_2_P are presented in Figure 11.

**Figure 12:**
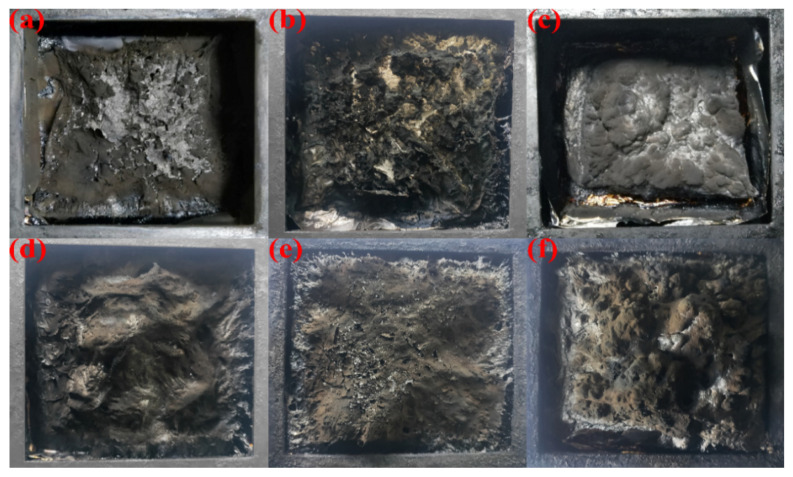
The digital photos of char residues after cone calorimeter test of neat RPUF (**a**), RPUF-MOF/MWCNTs 2 (**b**), RPUF-T (**c**), RPUF-T/MOF/MWCNTs 2 (**d**), RPUF-C-MOF/MWCNTs 2 (**e**), and RPUF-T/C-MOF/MWCNTs 2 (**f**).

From Figure 11a, the pHRR of RPUFs (except neat RPUF) were all lower than that of neat RPUF, which disclosed that the use of MOF/MWCNTs or C-MOF/MWCNTs or TBPBP decreased the pHRR and enhanced the fire safety during the combustion of RPUFs, especially the simultaneous use of C-MOF/MWCNTs and TBPBP. Notably, all HRR curves were not smooth and divided into several large and small peaks, which was caused by the combustion process of intermediary decomposition products, intermediary char, or volatiles [52,53,54]. THR curves of RPUFs were shown in Figure 11b. THR values of neat RPUF, RPUF-MOF/MWCNTs 2, RPUF-T, RPUF-T/MOF/MWCNTs 2, RPUF-C-MOF/MWCNTs 2, and RPUF-T/C-MOF/MWCNTs 2 were 30.54 MJ/m^2^, 28.78 MJ/m^2^, 20.67 MJ/m^2^, 19.84 MJ/m^2^, 26.79 MJ/m^2^, 15.89 MJ/m^2^, respectively. The THR values displayed three results. The first result was that both MOF/MWCNTs and C-MOF/MWCNTs slightly improved the fire safety by promoting the formation of a stable carbon layer. Second, TBPBP outstandingly strengthened the fire safety thanks to the formation of the intact, intumescent, sufficient char residues and the release of non-combustible gases (including CO_2_, NH_3_, H_2_O, phosphorus-containing gas, and so on) at high temperature [11]. The third result was that the simultaneous use of MOF/MWCNTs and TBPBP or the simultaneous use of C-MOF/MWCNTs and TBPBP both improved the fire resistance more effectively than the use of MOF/MWCNTs, C-MOF/MWCNTs, or TBPBP alone, due to their synergistic fire resistance. The specific synergistic fire resistance was that TBPBP released non-flammable gas and formed an expanded, complete, and stable carbon residue layer with C-MOF/MWCNTs or MOF/MWCNTs (seen Figure 12f and the residues in Table 4 below) because of the increase in viscosity of the carbon residue system, thereby diluting the oxygen concentration and preventing the transfer of oxygen and heat to the interior of RPUF. Meanwhile, the simultaneous use of C-MOF/MWCNTs and TBPBP was better than the simultaneous use of MOF/MWCNTs and TBPBP to improve the fire resistance of foams, because the MOF/MWCNTs continued to decompose in a fire (seen Figure S7), and the resulting char residues are less stable than that of the simultaneous use of C-MOF/MWCNTs and TBPBP (seen Figure S8).

In general, smoke is a crucial factor for influencing the evacuation in the fire accident and polluting the environment [55]. Smoke release during the combustion of RPUFs was recorded via SPR and TSP curves, which were presented in Figure 11c,d, respectively. Due to the early decomposition of TBPBP, RPUF-T, RPUF-T/MOF/MWCNTs 2, and RPUF-T/C-MOF/MWCNTs 2, the pSPR reached earlier than neat RPUF, RPUF-MOF/MWCNTs 2, and RPUF-C-MOF/MWCNTs 2. Thanks to the synergistic effect of C-MOF/MWCNTs and TBPBP, the pSPR of RPUF-C-MOF/MWCNTs 2 significantly decreased in comparison with RPUF-T, which revealed their simultaneous utilization enhanced the smoke safety during RPUFs’ combustion. From Figure 11d, RPUF-MOF/MWCNTs 2, RPUF-T, RPUF-T/MOF/MWCNTs 2, RPUF-C-MOF/MWCNTs 2, and RPUF-T/C-MOF/MWCNTs 2 all gained lower TSP values compared with neat RPUF, which decreased from 7.69 m^2^ to 7.64 m^2^, 5.07 m^2^, 4.56 m^2^, 6.79 m^2^, and 3.81 m^2^, respectively. Contrary to neat RPUF, the TSP value of RPUF-T/MOF/MWCNTs 2 and RPUF-T/C-MOF/MWCNTs 2 diminished by 40.70% and 50.46%, respectively. Evidently, it is most effective to improve smoke safety and abate environmental pollution when C-MOF/MWCNTs and TBPBP are simultaneously employed.

It is internationally conceded that the toxic CO comes from incomplete combustion of materials, and the catalytic conversion of CO to non-toxic CO_2_ is a common method to reduce CO concentration. COP curves and TCOP curves were shown in Figure 11e,f, respectively. From Figure 11e, the peak of COP of RPUF-T quickly achieved a fearsome 0.0126 g/s, which was because the formation of the intumescent and intact char residues that were promoted by the early decomposition of TBPBP obstructed the sufficient burning of the foam matrix. Meanwhile, the peak of COP of RPUF-T/MOF/MWCNTs 2 decreased to 0.0097 g/s, probably due to the conversion of CO to CO_2_ by the catalysis of CO_3_O_4_ and CuO derived from the thermal decomposition of MOF/MWCNTs in a fire. Notably, the peak of COP of RPUF-T/C-MOF/MWCNTs 2 only reached 0.0056 g/s because of the conversion of CO to CO_2_ by the catalysis of CO_3_O_4_ and CuO in C-MOF/MECNTs. Meanwhile, TCOP of RPUF-T/C-MOF/MWCNTs 2 (0.34 g) were remarkably reduced compared to these of RPUF-T (0.54 g) and RPUF-T/MOF/MWCNTs 2 (0.46 g). The above results disclosed that the introduction of C-MOF/MWCNTs 2 into RPUF obviously decreased the COP during the combustion of RPUF, effectively facilitating people’s escape.

CO_2_P curves (Figure 11g) and TCO_2_P curves (Figure 11h) reveal that compared with neat RPUF and RPUF-T, the use of MOF/MWCNTs or C-MOF/MWCNTs did not increase CO2P. The phenomenon could be caused by two reasons. On the one hand, although CO was catalyzed to CO_2_, the CO release was much lower than the CO_2_ release during the combustion of RPUFs. On the other hand, the employment of MOF/MWCNTs or C-MOF/MWCNTs improved the char residue stability of RPUFs, resulting in facilitating the conversion of some foam matrix to the char residues instead of CO_2_, which was confirmed by the digital photos of char residues after CC test of RPUFs in Figure 12 and the amount of char residues in Table 4. From Figure 12, compared to the char residue of RPUF-T, both RPUF-T/MOF/MWCNTs 2 and RPUF-T/C-MOF/MWCNTs 2 possessed the firmer, more holistic, and intumescent char residues because of the synergistic effect of MOF/MWCNTs and TBPBP or C-MOF/MWCNTs and TBPBP, which were consistent with the amount of char residues of RPUF-T (9.29 wt%), RPUF-T/MOF/MWCNTs 2 (11.83 wt%), and RPUF-T/C-MOF/MWCNTs 2 (14.18 wt%) in Table 4.

TTI value reveals whether the material is easily ignited. The larger TTI value means the better ignition resistance for materials. Both MOF/MWCNTs and C-MOF/MWCNTs failed to increase TTI values for RPUFs, agreeing with low LOI values and UL-94 ranks. While RPUF-T/MOF/MWCNTs 2 and RPUF-T/C-MOF/MWCNTs 2 had high TTI values like RPUF-T owing to the char-promoting effect of TBPBP, the synergistic effect of TBPBP and MOF/MWCNTs or C-MOF/MWCNTs. Av-EHC displays the degree of burning of volatile gases produced from the materials during combustion [56,57]. Av-EHC values of RPUF-MOF/MWCNTs 2 (20.23 MJ/kg) and RPUF-C-MOF/MWCNTs 2 (22.04 MJ/kg) were not changed much in contrast with that of neat RPUF (21.36 MJ/kg), disclosing that using MOF/MWCNTs or C-MOF/MWCNTs alone had a little effect on Av-EHC for RPUFs. Av-EHC values of RPUF-T, RPUF-T/MOF/MWCNTs 2 and RPUF-T/C-MOF/MWCNTs 2 decreased to 15.75 MJ/kg, 14.97 MJ/kg, and 13.26 MJ/kg, respectively, suggesting that RPUFs inserting TBPBP decomposed phosphorus-containing inhibitors to interrupt the combustion of RPUFs and generate the decrease of Av-EHC [11].

### 3.3. Mechanism for Enhancing Fire and Smoke Safety

**Scheme 3:**
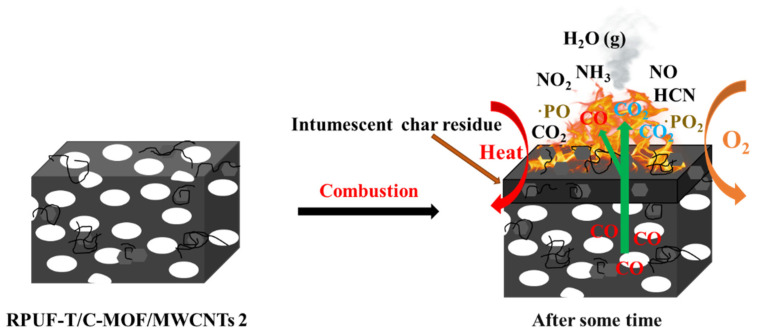
A possible mechanism for enhancing fire and smoke safety of RPUF-T/C-MOF/MWCNTs 2.

Among all RPUFs, RPUF-T/C-MOF/MWCNTs 2 achieved the best flame retardancy and smoke suppression owing to the synergistic effect of C-MOF/MWCNTs and TBPBP. Specifically, TBPBP released non-flammable gas at high temperature, including CO_2_, NH_3_, H_2_O, phosphorus-containing gas, and so on [11]; meanwhile, using TBPBP and C-MOF/MWCNTs promoted the formation of a firm, complete, intumescent, maximum amount of carbon residue layer with C-MOF/MWCNTs because of the increase in viscosity of carbon residue system, thereby diluting the oxygen concentration and preventing the transfer of oxygen and heat into the interior of RPUF. Thus, RPUF-T/C-MOF/MWCNTs 2 had the binary flame retardant mode of condensed phase flame retardant and gas phase flame retardant. Actually, RPUF-T/C-MOF/MWCNTs 2 gained an outstanding effect on reducing the amount of CO release, because CO_3_O_4_ and CuO catalyzed the conversion of CO to CO_2_ [58]. When CO_3_O_4_ and CuO catalyzed the conversion of CO into CO_2_, CO_3_O_4_ was converted into CoO, and CuO was converted into Cu. Under the action of high temperature and oxygen, CoO and Cu were again converted into CO_3_O_4_ and CuO for participating in the catalysis of CO. A possible mechanism for enhancing fire and smoke safety of RPUF-T/C-MOF/MWCNTs 2 are respectively shown in Scheme 3.

## 4. Conclusions

Compared to neat RPUF, RPUF-MOF/MWCNTs 2 and RPUF-C-MOF/MWCNTs 2 had a certain improvement in physical properties such as the compressive performance, thermal stability, etc., disclosing MOF/MWCNTs and C-MOF/MWCNTs all enhanced physical properties of RPUFs. However, RPUF-MOF/MWCNTs 2 and RPUF-C-MOF/MWCNTs 2 did not achieve good flame retardant properties, because none of them formed firm char residue layers, or generated inhibitory gases that were conducive to flame retardancy. Meanwhile, RPUF-MOF/MWCNTs 2 made no progress in reducing the release of COP in contrast to neat RPUF on account of less Co_3_O_4_, CuO created via MOF in MOF/MWCNTs during combustion. However, compared to RPUF-MOF/MWCNTs 2, RPUF-C-MOF/MWCNTs 2 possessed more Co_3_O_4_, CuO, resulting in the better improvement in suppressing the emission of THR, TSP, COP, and CO_2_P, especially the 55.17% COP drop compared to neat RPUF. RPUF-T/C-MOF/MWCNTs 2 gained the best flame retardancy, smoke suppression, CO emission reduction, as well as the best physical properties (including compressive properties, thermal stability, etc.) among all RPUFs because of the synergistic effect of TBPBP and C-MOF/MECNTs, providing a certain reference significance for fabricating RPUFs with human safety and environmental protection as building insulation materials.

## Figures and Tables

**Table 1:** The formulation of bio-based RPUFs.

Samples	MCOP	PEG-200	TBPBP	Deionized Water	DMP-30	Silicone Oil	MOF /MWCNTs	C-MOF /MWCNTs	pMDI
neat RPUF [11]	80	20	0	4	2	4	0	0	146.79
RPUF-MOF/MWCNTs 1	80	20	0	4	2	4	5	0	146.79
RPUF-MOF/MWCNTs 2	80	20	0	4	2	4	10	0	146.79
RPUF-MOF/MWCNTs 3	80	20	0	4	2	4	15	0	146.79
RPUF-T [11]	35	20	45	4	2	4	0	0	154.91
RPUF-T/MOF/MWCNTs 2	35	20	45	4	2	4	10	0	154.91
RPUF-MOF/C-MWCNTs 2	80	20	0	4	2	4	0	10	146.79
RPUF-T/C-MOF/MWCNTs 2	35	20	45	4	2	4	0	10	154.91

Note: 1. Neat RPUF and RPUF-T, respectively, refer to neat RPUF and RPUF-T45 in our previous work [11]. 2. Neat RPUF and RPUF-T are introduced to systematically study the effects of MOF/MWCNTs and C-MOF/MWCNTs on the physical properties, flame retardancy, and smoke suppression of bio-based RPUFs. 3. The unit of all reagents is gram.

**Table 2:** Density, compressive strength, and thermal conduction coefficient of RPUFs.

Sample	Density (kg/m^3^)	Compressive Strength (kPa)	Thermal Conductivity Coefficient W/(mK)
neat RPUF [11]	36.7 ± 0.9	236 ± 29	31.67 × 10^−3^
RPUF-MOF/MWCNTs 1	37.6 ± 0.5	301 ± 26	33.26 × 10^−3^
RPUF-MOF/MWCNTs 2	38.5 ± 0.6	358 ± 17	34.01 × 10^−3^
RPUF-MOF/MWCNTs 3	39.0 ± 0.8	379 ± 28	34.29 × 10^−3^
RPUF-T [11]	38.2 ± 0.7	422 ± 30	34.65 × 10^−3^
RPUF-T/MOF/MWCNTs 2	40.9 ± 1.1	453 ± 39	35.53 × 10^−3^
RPUF-C-MOF/MWCNTs 2	38.7 ± 0.9	367 ± 23	33.88 × 10^−3^
RPUF-T/C-MOF/MWCNTs 2	40.6±1.3	486 ± 25	34.93 × 10^−3^

**Table 3:** LOI values and UL-94 ranks of RPUFs.

Samples	LOI (%)	UL-94
neat RPUF	19.2	N.R.
RPUF-MOF/MWCNTs 1	19.6	N.R.
RPUF-MOF/MWCNTs 2	20.1	N.R.
RPUF-MOF/MWCNTs 3	20.4	N.R.
RPUF-T	25.6	V-0
RPUF-T/MOF/MWCNTs 2	27.4	V-0
RPUF-C-MOF/MWCNTs 2	20.3	N.R.
RPUF-T/C-MOF/MWCNTs 2	28.1	V-0

Note: 1. LOI value is a mean value of parallel tests for the same RPUF. 2. N.R. refers to no rank.

**Table 4:** Characteristic CC data of the chosen RPUFs.

Samples	TTI	pHHR	THR	pSPR	TSP	Av-EHC	Residue
	s	kW/m^2^	MJ/m^2^	m^2^/s	m^2^	MJ/kg	wt%
neat RPUF	0	315.56	30.54	0.096	7.69	21.36	3.17
RPUF-MOF/MWCNTs 2	0	261.76	28.78	0.078	7.64	20.23	4.06
RPUF-T	3	287.69	20.67	0.119	5.07	15.75	9.29
RPUF-T/MOF/MWCNTs 2	3	246.15	19.84	0.092	4.56	14.97	11.83
RPUF-C-MOF/MWCNTs 2	0	242.94	26.79	0.071	6.79	22.04	5.26
RPUF-T/C-MOF/MWCNTs 2	4	165.55	15.89	0.059	3.81	13.26	14.18

## Data Availability

Not applicable.

## Supplementary Materials

The following supporting information can be downloaded at: https://www.mdpi.com/article/10.3390/polym14173630/s1, Figure S1: FT-IR spectrum (a) and XRD curve (b) of MOF/MWCNTs; Figure S2: XPS curves of MOF/MWCNTs (a), its Co2p (b) and Cu2p (c); Figure S3: FSEM images of MOF/MWCNTs at different magnifications; Figure S4: FSEM image of MOF/MWCNTs with mapping mode (a),EDXS elemental mapping of MOF/MWCNTs ((b): C, (c): N, (d): O, (e): Co, (f): Cu); Figure S5: TEM images (a), (b) of MOF/MWCNTs; Figure S6: TEM image of MOF with mapping mode (a), EDXS elemental mapping of MOF ((b): C, (c): N, (d): O, (e): Co, (f): Cu), EDXS curves of MOF (g); Figure S7: TGA curves (a) under N_2_ and air, DTG curve under N_2_ (b) and DTG curve under air (c) of MOF/MWCNTs; Figure S8: Raman curves of char residues of RPUFs; Figure S9: FT–IR spectrum (a), XRD curve (b), Raman curve (c) of B-MOF/MWCNTs; Figure S10: XPS curves of B-MOF/MWCNTs (a), its Co2p (b) and Cu2p (c); Figure S11: FSEM images (a, b, c) and TEM images (d, e) of C-MOF/MWCNTs at different magnifications, SAED pattern of B-MOF in B-MOF/MWCNTs (f); Figure S12: FSEM image of C-MOF/MWCNTs with mapping mode (a), EDXS elemental mapping of C-MOF/MWCNTs ((b): C, (c): N, (d): O, (e): Co, (f): Cu); Figure S13: TEM image of C-MOF with mapping mode (a), EDXS elemental mapping of C-MOF ((b): C, (c): N, (d): O, (e): Co, (f): Cu), EDXS curves of C-MOF (g); Figure S14: FT-IR spectrum (a), XRD curve (c) of char residue of RPUF-MOF/MWCNTs 2, FT-IR spectrum (b), XRD curve (d) of char residue of RPUF-T/MOF/MWCNTs 2, Raman spectra (e) of char residues of RPUF-MOF/MWCNTs 2 and RPUF-T/MOF/MWCNTs 2; Figure S15: FSEM images (a, b), TEM image (e), and SAED pattern (f) of char residue of RPUF-MOF/MWCNTs 2, FSEM images (c, d), TEM image (g), and SAED pattern (h) of char residue of RPUF-T/MOF/MWCNTs 2; Figure S16: FT-IR spectrum (a), XRD curve (c) of char residue of RPUF-C-MOF/MWCNTs 2, FT-IR spectrum (b), XRD curve (d) of char residue of RPUF-T/C-MOF/MWCNTs 2, Raman spectra (e) of char residues of RPUF-C-MOF/MWCNTs 2 and RPUF-T/C-MOF/MWCNTs 2; Figure S17: FSEM images (a, b), TEM image (e), and SAED pattern (f) of char residue of RPUF-C-MOF/MWCNTs 2, FSEM images (c, d), TEM image (g), and SAED pattern (h) of char residue of RPUF-T/C-MOF/MWCNTs 2; Table S1: The mass ratio and atom ratio of elements of FSEM image of MOF/MWCNTs with mapping mode; Table S2: The mass ratio and atom ratio of elements of TEM image of MOF with mapping mode; Table S3: The proportion of elements of XPS curves of MOF/MWCNTs and C-MOF/MWCNTs; Table S4: The mass ratio and atom ratio of elements of FSEM image of C-MOF/MWCNTs with mapping mode; Table S5: The mass ratio and atom ratio of elements of TEM image of C-MOF with mapping mode; Table S6: TGA and DTG data of RPUFs under N2; Table S7: TGA and DTG data of RPUFs under air; Table S8: The proportion of elements of MOF/MWCNTs and B-MOF/MWCNTs. Results and Discussion of MOF/MWCNTs, Laser Raman Spectroscopy Tests of Char Residues and the rest of possible flame-retardant and catalytic CO mechanism are presented in Supplementary Materials.
