# Supplementary material for: Bio-Based Rigid Polyurethane Foams Modified with C-MOF/MWCNTs and TBPBP as Building Insulation Materials: Synergistic Effect and Corresponding Mechanism for Enhancing Fire and Smoke Safety
Source: Polymers (Basel). 2022 Sep 2;14(17):3630. doi: 10.3390/polym14173630 (PMC9459931; doi:10.3390/polym14173630)
Supplement: Supplementary file 1 [file polymers-14-03630-s001.zip › polymers-1892023-supplementary.pdf]

Bio-based rigid polyurethane foams modified with C-MOF/MWCNTs and TBPBP as building insulation materials: Synergistic effect and corresponding mechanism for enhancing fire and smoke safety

Guangxu Bo[1], Xiaoling Xu[1], Xiaoke Tian, Jinyong Yan*, Xingjian Su, Yunjun Yan*

Key Laboratory of Molecular Biophysics of the Ministry of Education, College of Life Science and Technology, Huazhong University of Science and Technology, Wuhan 430074, China.

[1] These authors contributed equally to this work.

[*] Corresponding author. E-mail address: yjiny@126.com (Jinyong Yan), yanyunjun@hust.edu.cn (Yunjun Yan)

1. Results and Discussion

1.1. Characterization of MOF/MWCNTs

Before MOF/MWCNTs was used for preparing RPUFs, the corresponding physical and chemical properties had to test in order to ensure its successful synthesis and effective application. Fourier-transform infrared (FT-IR) spectrum and X-ray diffraction (XRD) curve of MOF/MWCNTs were exhibited in Figure S1a and S1b, respectively. From Figure S1a, vital peaks at 3421 cm^{-1}, 2927 cm^{-1} and 1584 cm^{-1} were disclosed, which were respectively assigned as the stretching vibrations of N-H, $-CH_3$ and C=N of imidazole ring on 2-MIM. Meanwhile, the sharp peak at 424 cm^{-1} of the stretching vibration of Co-N was found [1,2]. The absorption peak at 600-1500 cm^{-1} were associated with the stretching and bending vibration of the imidazole ring. Specifically, the intense and convoluted bands at 1350-1500 cm^{-1} was caused by the entire ring stretching, and the bands in the spectral region of 900-1350 cm^{-1} were ascribed for the in-plane bending of the ring, while those below 800 cm^{-1} were attributed to out-of-plane bending [2-4]. From Figure S1b, many critical patterns of ZIF-67 (a major constituent of MOF/MWCNTs) were found, including 7.15° (011), 10.19° (002), 12.53° (112), 14.54° (022), 16.26° (013), 17.83° (222), 21.93° (114), 24.30° (233), 25.41° (224), 26.47° (134), 29.47° (044), 30.37° (334), 31.29° (244) and 32.16° (235). These peak positions were consistent with those of the reported literatures [5,6].

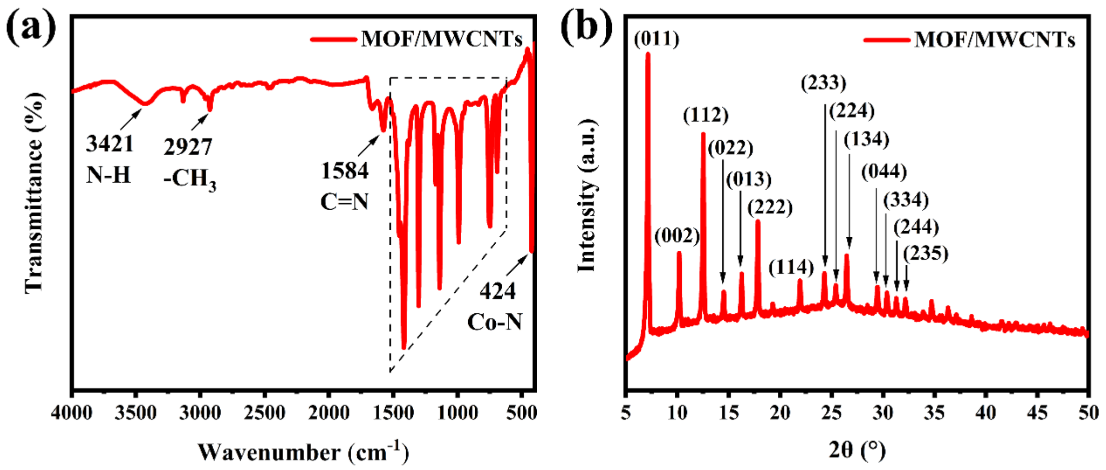

Figure S1. FT-IR spectrum (a) and XRD curve (b) of MOF/MWCNTs.

The peaks of MOF/MWCNTs, its Co2p and Cu2p in X-ray photoelectron spectroscopy (XPS) curves were all detected in Figure S2. In Co2p spectrum, there are four peaks, namely around appearing at 780.9 eV, 786.6 eV, 797.0 eV and 801.8 eV, corresponding to $Co2p_{3/2}$, the satellite peak of $Co2p_{3/2}$, $Co2p_{1/2}$, and the satellite peak of $Co2p_{1/2}$. Notably, the difference between the peaks of $Co2p_{3/2}$ and $Co2p_{1/2}$ was ca.16.1 eV, corresponding to the published literature [6]. It is unfortunate that Cu2p spectrum (Fig. S2c) wasn't well fitted, which was probably due to the presence of less Cu^{2+} via partially replacing Co^{2+} sites in the framework of ZIF-67.

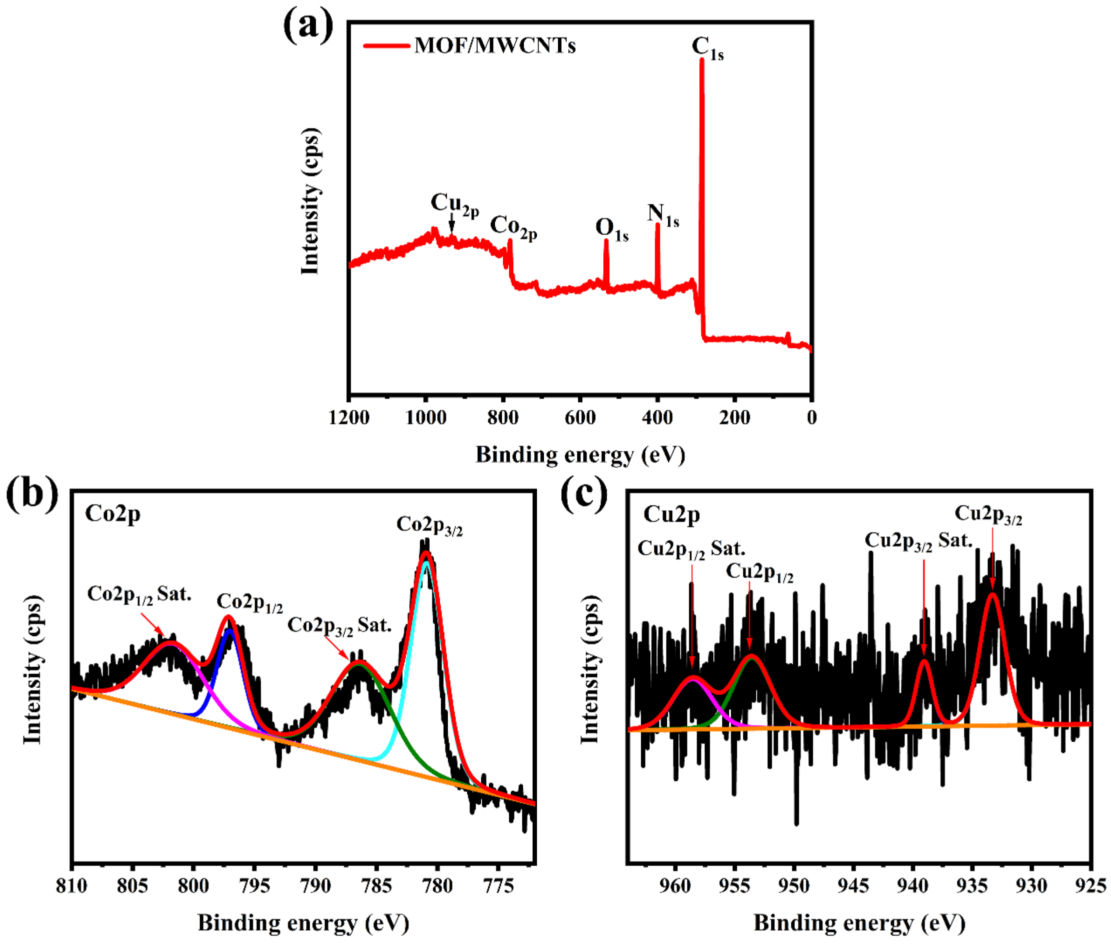

Figure S2. XPS curves of MOF/MWCNTs (a), its Co2p (b) and Cu2p (c).

Field emission scanning electron microscope (FSME) images of MOF/MWCNTs were presented in Figure S3. From Figure S3, MOF/MWCNTs consisted of MOF (dodecahedral structure) and MWCNTs, and the size of MOF was ca.1-2 um, and the vast majority of MOF was embedded in MWCNTs, a part of MOF was clustered together, and a very small number of MOF was distributed separately. The presence of copper ions did not change the framework of ZIF-67, which remained regular dodecahedron structure. As shown in Figure S3d, some nanotubes were adhered to the surface of MOF. Corresponding to FSEM image with mapping mode and specific energy dispersive X-ray spectroscopy (EDXS) elemental mapping of MOF/MWCNTs

were showed in Figure S4. And The mass ratio and atomic number ratio of elements of MOF/MWCNTs were listed in Table S1. Figure S4 and Table S1 disclose the presence of elements (C, N, O, Co, Cu) of MOF/MWCNTs. The mass ratio and atom ratio of Cu were obviously lower than these of Co, which were broadly in line with those of its FT-IR spectrum, XRD and XPS curves.

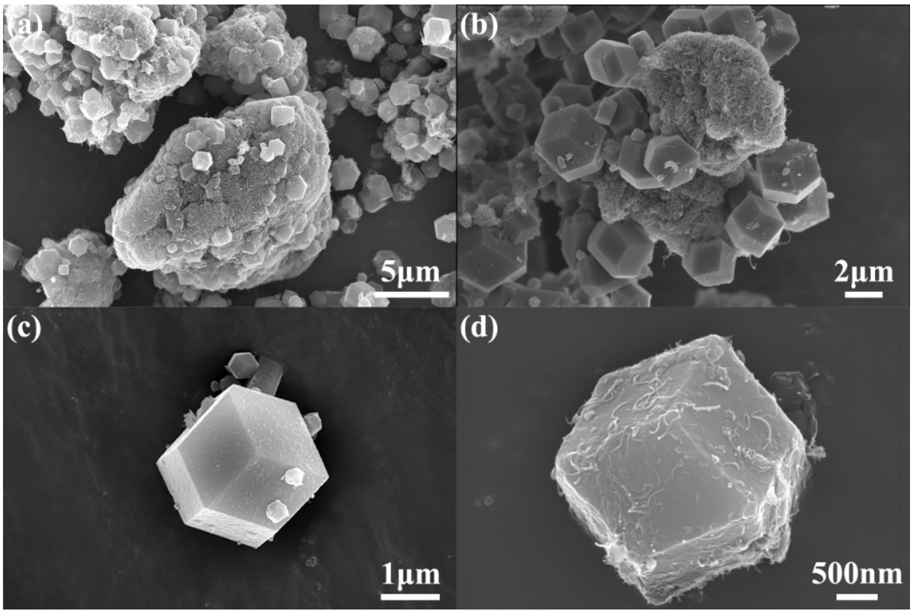

Figure S3. FSEM images of MOF/MWCNTs at different magnifications.

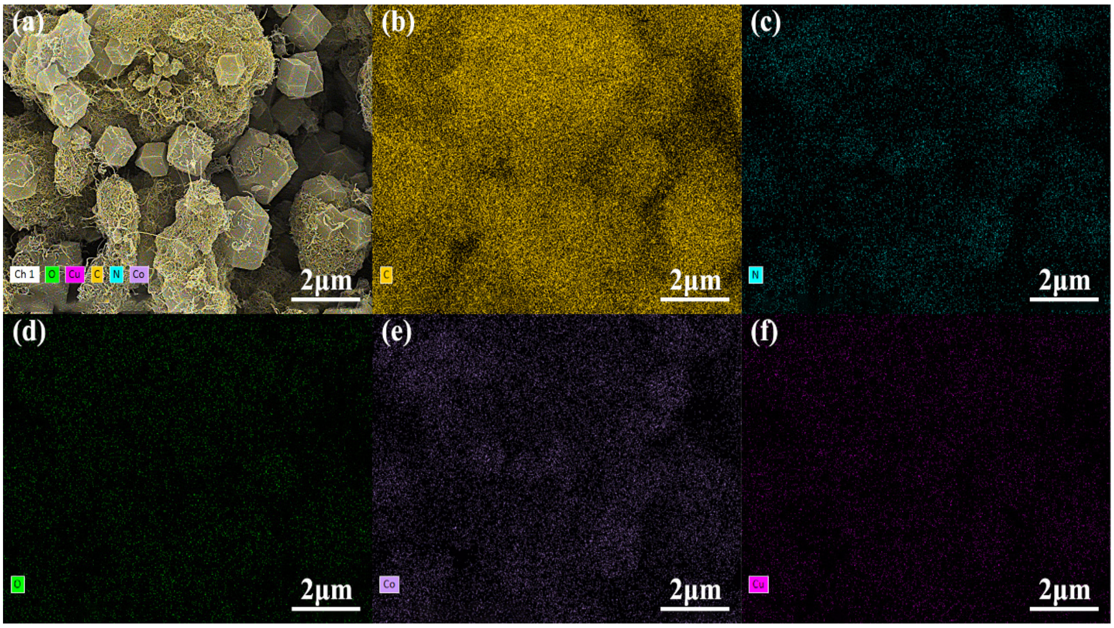

Figure S4. FSEM image of MOF/MWCNTs with mapping mode (a),EDXS elemental

mapping of MOF/MWCNTs ((b):C, (c):N, (d):O, (e):Co, (f):Cu).

Table S1. The mass ratio and atom ratio of elements of FSEM image of MOF/MWCNTs with mapping mode.

Element	Mass (wt%)	Atom (%)
C	65.25	74.30
N	20.18	19.70
O	4.24	3.62
Co	8.89	2.06
Cu	1.43	0.31

Transmission electron microscopy (TEM) images (Fig. S5a and S5b) exhibit MOF possessed dodecahedral structure wrapped around MWCNTs, which verified almost the same results as those of FSEM images. TEM image with mapping mode and specific EDXS elemental mapping of MOF, the main composited part of MOF/MWCNTs, were showed in Figure S6, and the mass ratio and atomic number ratio of elements of MOF were showed in Table S2. Particularly worth mentioning was that Figure S6 and Table S2 also proved that partial Cu^{2+} replaced some Co^{2+} sites in the host framework of ZIF-67 for forming MOF.

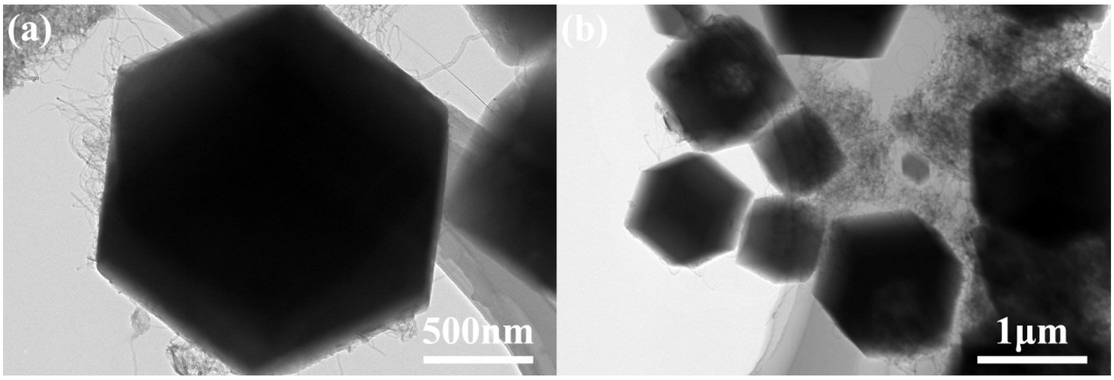

Figure S5. TEM images (a), (b) of MOF/MWCNTs.

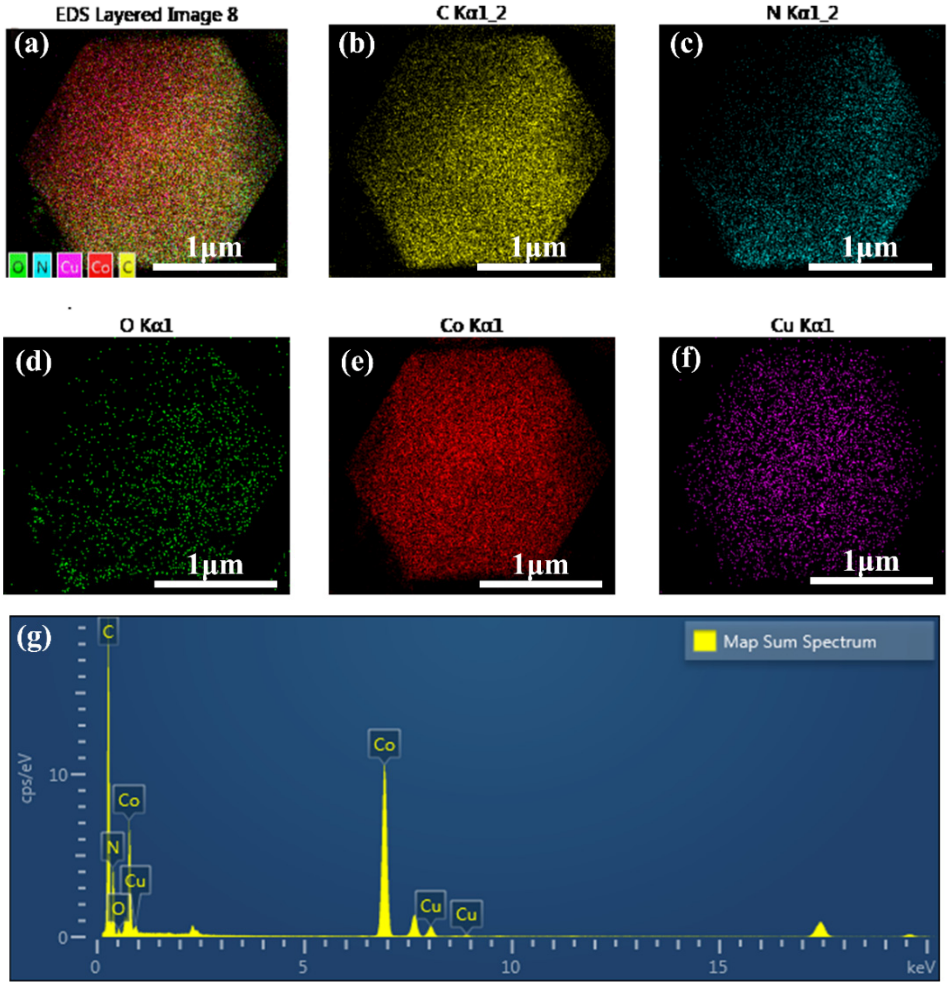

Figure S6. TEM image of MOF with mapping mode (a), EDXS elemental mapping of MOF ((b):C, (c):N, (d):O, (e):Co, (f):Cu), EDXS curves of MOF (g).

Table S2. The mass ratio and atom ratio of elements of TEM image of MOF with mapping mode.

Element	Mass (wt%)	Atom (%)
C	47.51	70.61
N	13.41	17.08
O	0.63	0.71
Co	37.53	11.34
Cu	0.92	0.26

The results of above characterization indicate that MOF/MWCNTs was successfully prepared.

To investigate thermal stability of MOF/MWCNTs, thermogravimetric analysis (TGA) was performed. Corresponding to TGA curves, DTG curves under N_2 and air were displayed in Figure S7. Figure S7a shows that the residue of MOF/MWCNTs were respective 54.12 wt% and 26.20 wt% under N_2 and air at 700 °C, and MOF/MWCNTs respectively underwent a main thermal decomposition at 500-650 °C and 305-315 °C due to the thermolysis of organic ligands (2-MIM). While a main thermal decomposition under air was more intense and took less time than that under N_2. Furtherly, compared to DTG curve under N_2 (Fig. S7b), DTG curve under air (Fig. S7c) also confirmed that there was a more intense and less time consuming main thermal decomposition because of the oxidative decomposition of organic ligands in MOF/MWCNTs. The results evince MOF/MWCNTs possessed the better thermal

stability under N_2 than that of under air.

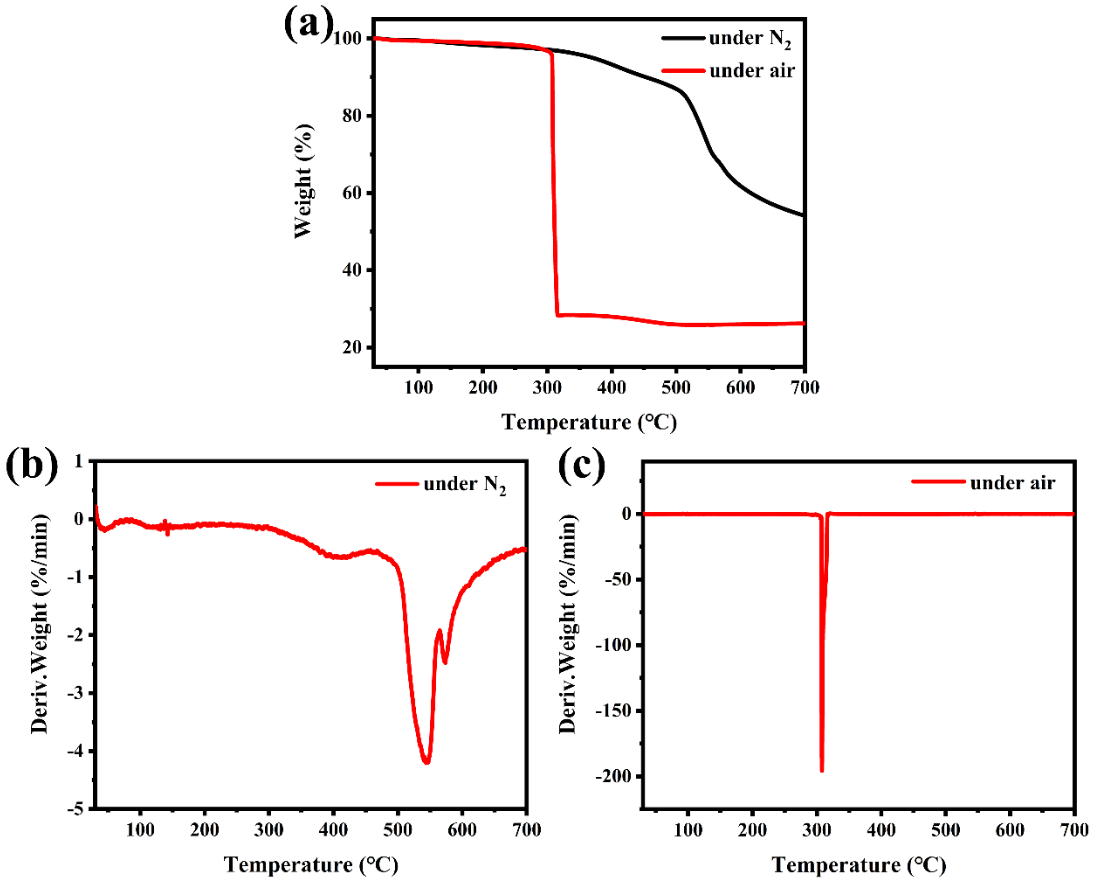

Figure S7. TGA curves (a) under N_2 and air, DTG curve under N_2 (b) and DTG curve under air (c) of MOF/MWCNTs.

1.2. Characterization of RPUFs

1.2.1. Laser Raman Spectroscopy Tests of Char Residues

Laser Raman spectroscopy test is a common method for evaluating the stability of carbonaceous chars on the basis of the graphitization degree (or structural disorder degree) [7,8]. The graphitization degree is characterized via the intensity ratio of D/G bands (I_D/I_G), and the lower ratio of I_D/I_G, the more stable structure of the char residue is [9]. D (or “Defect”) band at ca.1360 cm^{-1} and G (or “Graphite”) band at ca.1580 cm^{-1}

that were showed in Figure S8 were respectively assigned to amorphous carbon (or disordered graphite or glassy carbon) and graphitized carbon [8-10]. As presented in Figure S8, I_D/I_G of neat RPUF, RPUF-MOF/MWCNTs 1, RPUF-MOF/MWCNTs 2, RPUF-MOF/MWCNTs 3, RPUF-T, RPUF-T/MOF/MWCNTs 2, RPUF-C-MOF/MWCNTs 2 and RPUF-T/C-MOF/MWCNTs 2 were respectively 3.47, 3.41, 3.36, 3.32, 3.23, 3.17, 3.29 and 3.02. Contrary to neat RPUF, the increasing use of MOF/MWCNTs improved the stability of char residues for RPUFs. C-MOF/MWCNTs enhanced the char residue stability of RPUF more than MOF/MWCNTs with the same added mass. However, MOF/MWCNTs or C-MOF/MWCNTs was both far less effective than TBPBP in enhancing the char residues' stability. Most notably, RPUF-T/C-MOF/MWCNTs 2 obtained the most stable char residues among all RPUFs, which was considered to the synergistic flame retardant effect of C-MOF/MWCNTs and TBPBP. These results of Laser Raman spectroscopy test were in agreement with those of vertical burning test, limiting oxygen index (LOI) test and FSEM test.

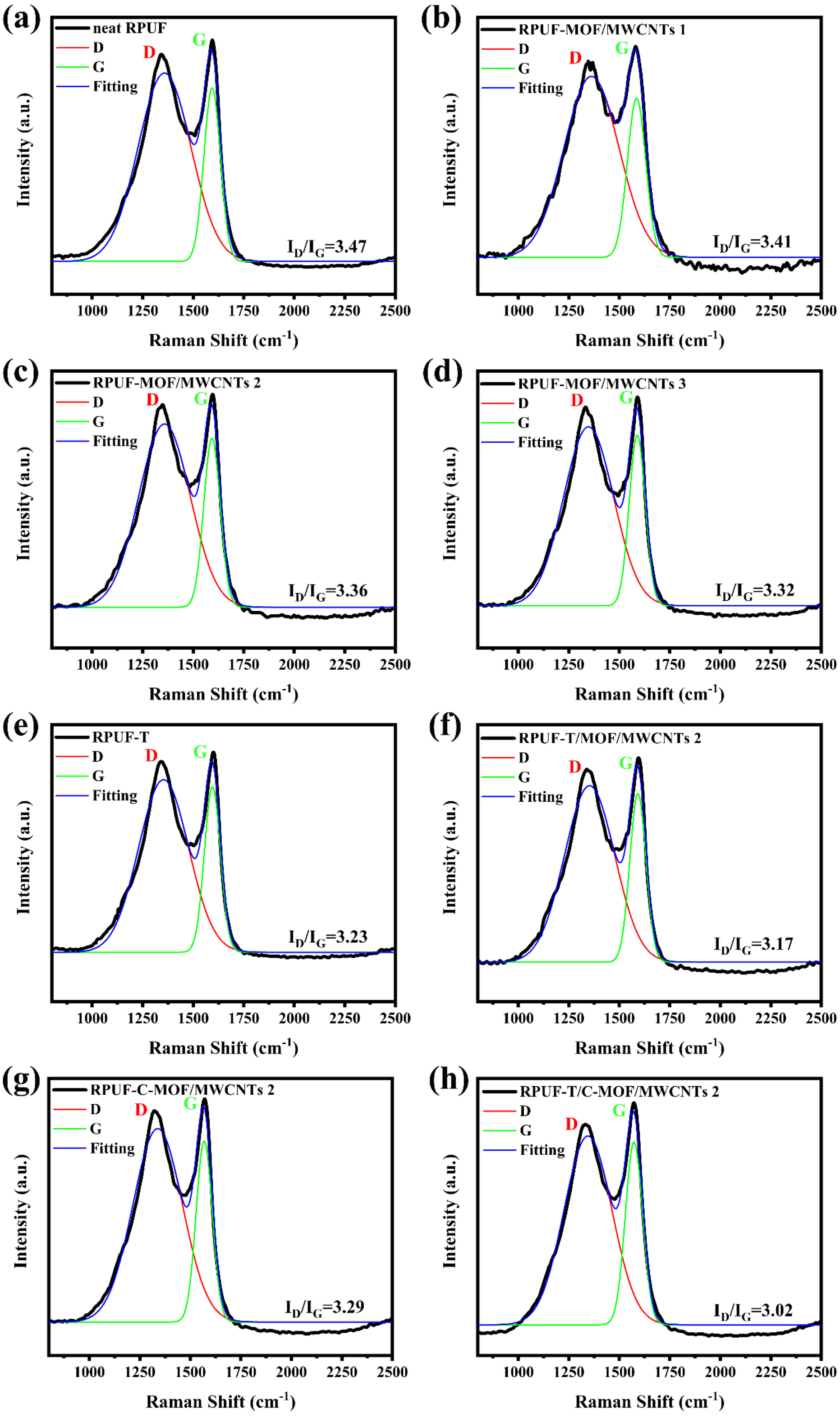

Figure S8. Raman curves of char residues of RPUFs.

1.2.2. Mechanism for enhancing fire and smoke safety

FT-IR spectrum, XRD curve and Laser Raman spectrum of B-MOF/MWCNTs were respectively presented in Figure S9a, S9b and S9c. FT-IR spectrum, XRD curve and Raman spectrum of B-MOF/MWCNTs were similar to these of C-MOF/MWCNTs, indicating that the main component of B-MOF/MWCNTs were Co_3O_4 and MWCNTs, which was consistent with these of C-MOF/MWCNTs. But the characteristic peaks of MOF/MWCNTs were still found, such as the stretching vibration peak of C=N of imidazole ring on 2-MIM at 1584 cm^{-1}, the peak at 424 cm^{-1} of the stretching vibration of Co-N, the absorption peak at 600-1500 cm^{-1} of the stretching and bending vibration of the imidazole ring, critical patterns (011) at 7.15° of MOF/MWCNTs. These peaks were discovered, revealing a small portion of MOF/MWCNTs was remained.

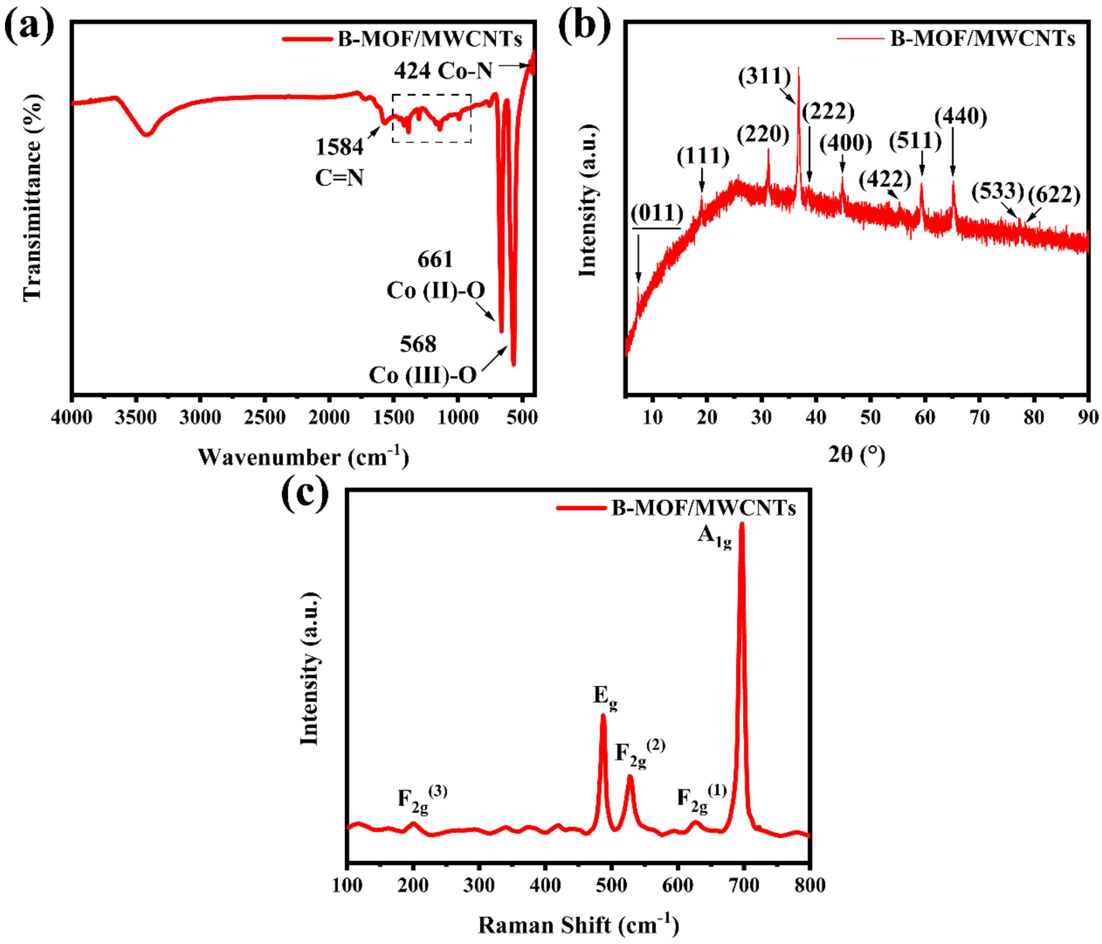

Figure S9. FT–IR spectrum (a), XRD curve (b), Raman curve (c) of B-MOF/MWCNTs.

XPS curves of B-MOF/MWCNTs, its Co2p curve and Cu2p curve were present in Figure S10a, S10b and S10c. These three curves and the proportion of elements (Table S8) were highly consistent with these of C-MOF/MWCNTs, which also displayed that B-MOF/MWCNTs were composed of a large amount of Co_3O_4, MWCNTs and a small amount of CuO.

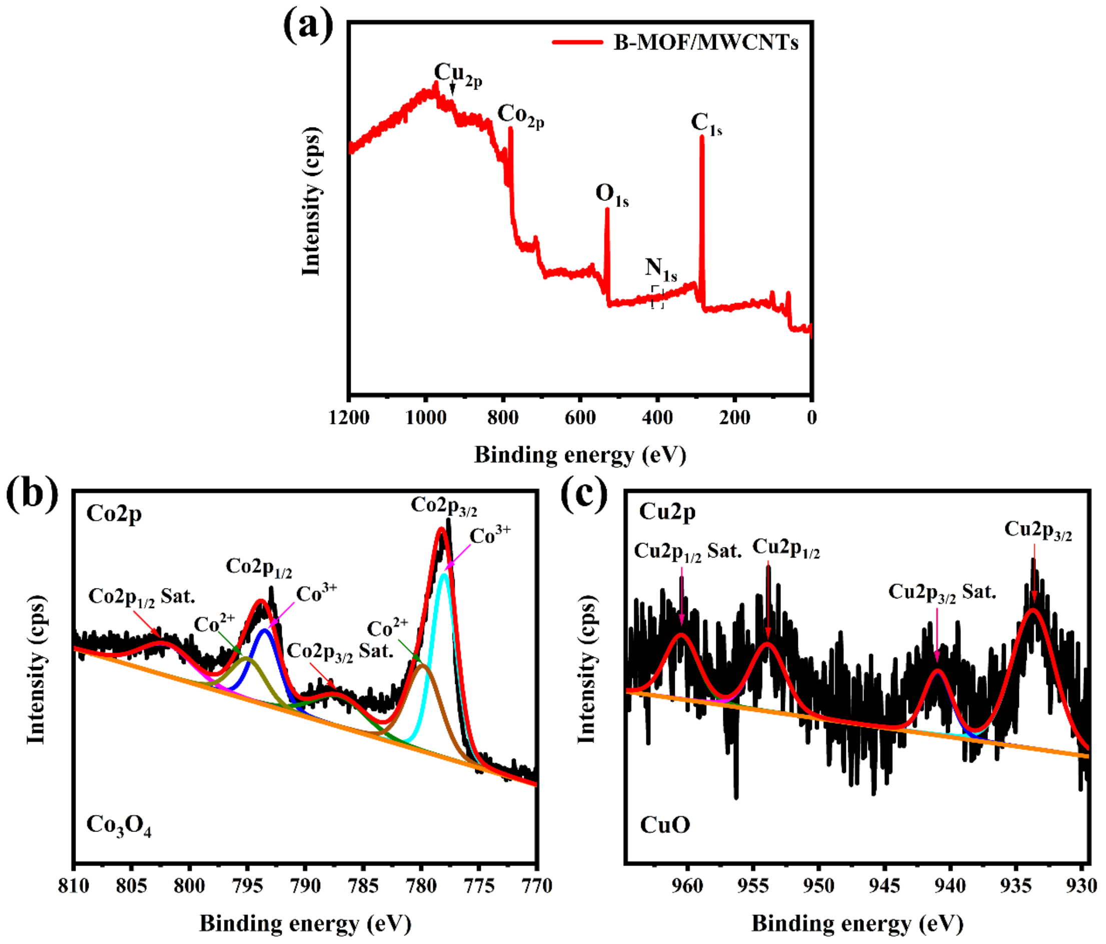

Figure S10. XPS curves of B-MOF/MWCNTs (a), its Co2p (b) and Cu2p (c).

FSEM images (Fig. S11a, S11b and S11c), TEM images (Fig. S11d and S11e) of B-MOF/MWCNTs exhibit three structures, namely MWCNTs, porous dodecahedrons and collapsed dodecahedrons. The porous decahedrons consisted of a large amount of Co_3O_4 and a small amount of CuO, which were proved via XPS curves of B-MOF/MWCNTs as mentioned above. Collapsed dodecahedrons were in a state of transition from formal dodecahedrons into porous dodecahedrons, and the main component of collapsed dodecahedrons were still ZIF-67 partially replaced copper ions. SAED pattern of B-MOF in B-MOF/MWCNTs (Fig. S11f) depicted the polycrystalline properties of Co_3O_4.

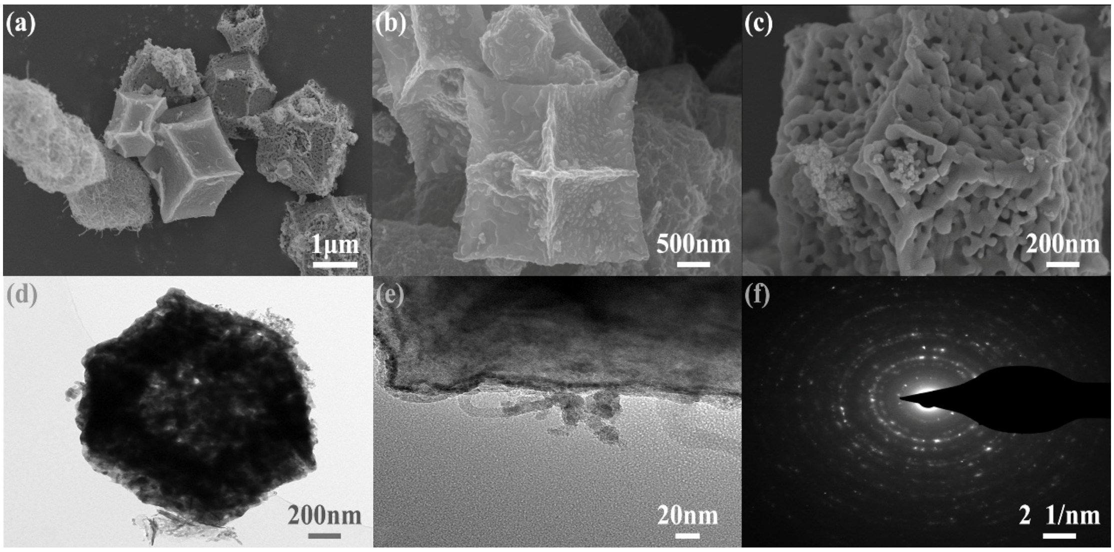

Figure S11. FSEM images (a, b, c) and TEM images (d, e) of C-MOF/MWCNTs at different magnifications, SAED pattern of B-MOF in B-MOF/MWCNTs (f).

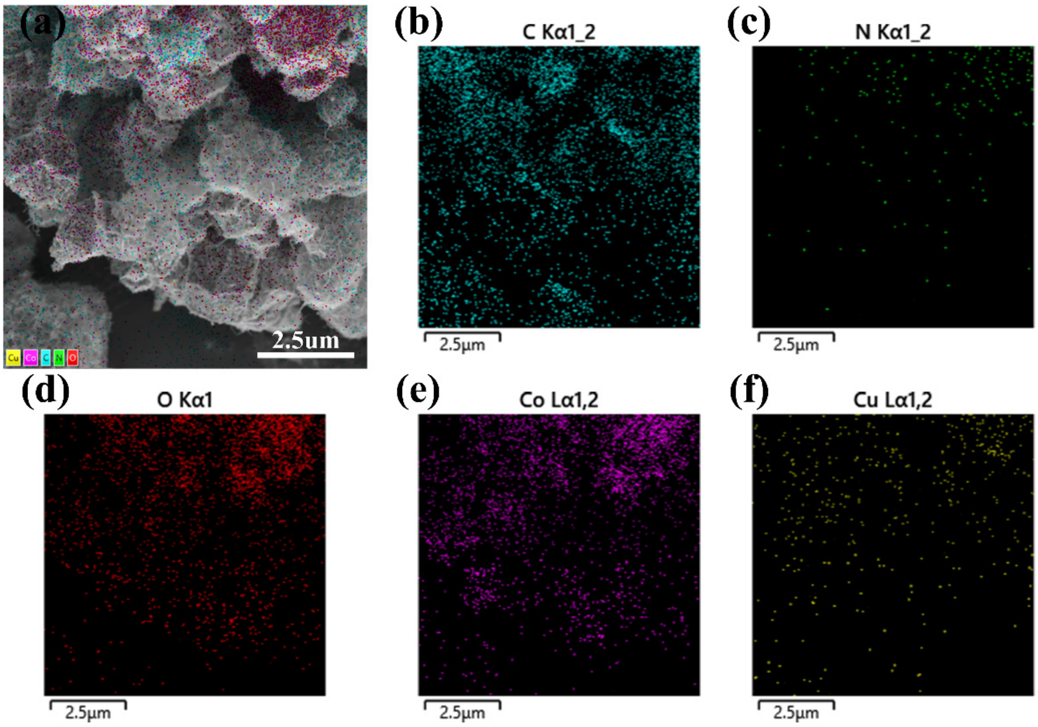

Figure S12. FSEM image of C-MOF/MWCNTs with mapping mode (a), EDXS elemental mapping of C-MOF/MWCNTs ((b):C, (c):N, (d):O, (e):Co, (f):Cu).

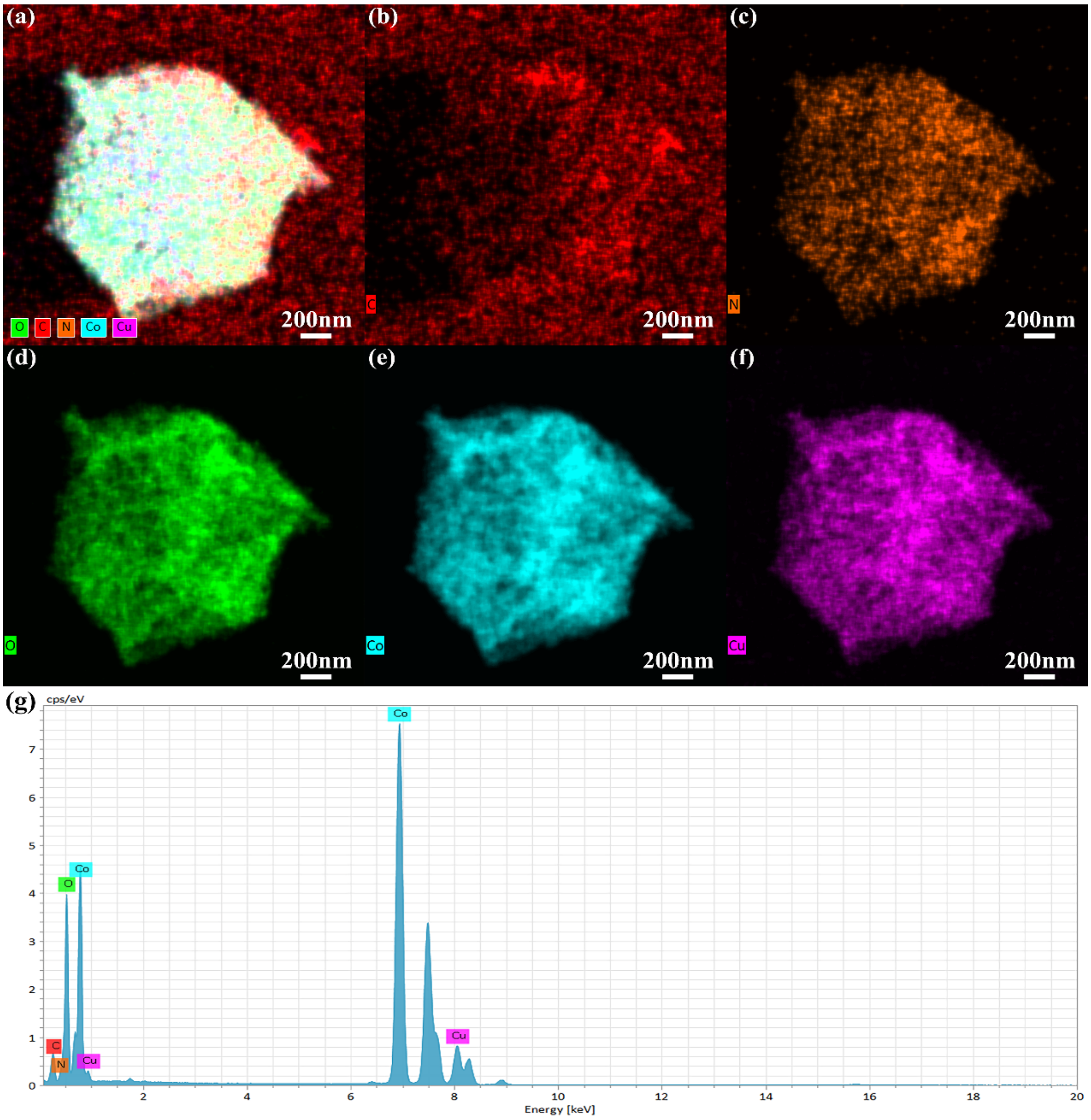

Figure S13. TEM image of C-MOF with mapping mode (a), EDXS elemental mapping of C-MOF ((b):C, (c):N, (d):O, (e):Co, (f):Cu), EDXS curves of C-MOF (g).

Based on the above characterization analyses, both RPUF-MOF/MWCNTs 2 and RPUF-C-MOF/MWCNTs 2 failed to effectively improve the flame retardant performance, because none of them formed a firm, complete char residue layer, and generated flame inhibitory gases. However, compared to RPUF-T, both RPUF-T/MOF/MWCNTs 2 and RPUF-T/C-MOF/MWCNTs 2 achieved the cracking flame retardancy, smoke suppression owing to the synergistic effect of MOF/MWCNTs and

TBPBP or C-MOF/MWCNTs and TBPBP resulting in a firm, complete, intumescent char residue layer and phosphorus-containing inhibitory gases, especially RPUF-T/C-MOF/MWCNTs 2. Notably, RPUF-MOF/MWCNTs 2 and RPUF-T/MOF/MWCNTs 2 almost failed to decrease the amount of CO release, while RPUF-C-MOF/MWCNTs 2 and RPUF-T/C-MOF/MWCNTs 2 all gained a outstanding effect on reducing the amount of CO release, disclosing that C-MOF/MWCNTs was more effective than MOF/MWCNTs in catalyzing CO to CO_2 and better environmental protection.

Before systematically studying the catalytic mechanism of CO, MOF/MWCNTs were placed in a crucible and then burned under an alcohol lamp for 60 s in order to simulate the combustion of MOF/MWCNTs in RPUFs. Burned MOF/MWCNTs was named as B-MOF/MWCNTs. B-MOF/MWCNTs was characterized via FT-IR, XRD, Laser Raman, XPS, FSEM and TEM. Corresponding Figures and Table (including Fig. S9-11, Table S8) and characterization analysis displays that the majority of MOF in B-MOF/MWCNTs was converted to Co_3O_4 and CuO, a small amount of MOF was retained. In fact, it was CO_3O_4 and CuO that catalyzed the conversion of CO to CO_2, rather than MOF. When CO_3O_4 and CuO catalyzed the conversion of CO into CO_2, CO_3O_4 was converted into CoO, and CuO was converted into Cu. Under the action of high temperature and oxygen, CoO and Cu were again converted into CO_3O_4 and CuO for participating in the catalysis of CO. In the simulated situation, a part of MOF was not converted into CO_3O_4 and CuO, and the conversion rate of MOF in the foam was possibly even lower. Therefore, adding the same mass of C-MOF/MWCNTs possessed

more CO_3O_4 and CuO than MOF/MWCNTs produced in foams, explaining that RPUF-C-MOF/MWCNTs 2 and RPUF-T/C-MOF/MWCNTs 2 had the better catalytic effect on CO than RPUF-MOF/MWCNTs 2 and RPUF-T/MOF/ MWCNTs 2.

In order to further investigate the catalytic mechanism of CO and verify the above conclusion, char residues of RPUF-MOF/MWCNTs 2, RPUF-T/MOF/MWCNTs 2, RPUF-C-MOF/MWCNTs 2 and RPUF-T/C-MOF/MWCNTs 2 were characterized via FT-IR, XRD, Laser Raman, FSEM and TEM. FT-IR spectra, XRD curves and Raman spectra of char residues of RPUF-MOF/MWCNTs 2, RPUF-T/MOF/MWCNTs 2 were presented in Figure S14. From FT-IR spectra and XRD curves, weak peaks of Co_3O_4 and CuO were detected, moreover, no characteristic peaks of Co_3O_4 were found in Raman spectra. FSEM images, TEM images and SAED patterns of RPUF-MOF/MWCNTs 2, RPUF-T/MOF/MWCNTs 2 were showed in Figure S15. As presented in Figure S15, MWCNTs, burned MOF (namely Co_3O_4 and CuO) and unburned MOF were all discovered. FT-IR spectra, XRD curves and Raman spectra of RPUF-MOF/C-MWCNTs 2 and RPUF-T/C-MOF/MWCNTs 2 (Fig. S16) all detected the distinct characteristic peaks of Co_3O_4 and CuO. The FSEM images, TEM images and SAED patterns (Fig. S17) also were detected in the presence of MWCNTs, C-MOF (i.e.Co_3O_4 and CuO). Characteristic results of char residues of RPUF-MOF/MWCNTs 2, RPUF-T/MOF/MWCNTs 2, RPUF-C-MOF/MWCNTs 2 and RPUF-T/C-MOF/MWCNTs 2 proved adding the same mass of C-MOF/MWCNTs possessed more CO_3O_4 and CuO than MOF/MWCNTs produced in foams, which was consistent with

COP curves and TCOP curves in CC test.

In summary, the possible flame-retardant and catalytic CO mechanism of RPUF-T/MOF/MWCNTs 2 and RPUF-T/C-MOF/MWCNTs 2 are respectively shown in Scheme S1a and 1b.

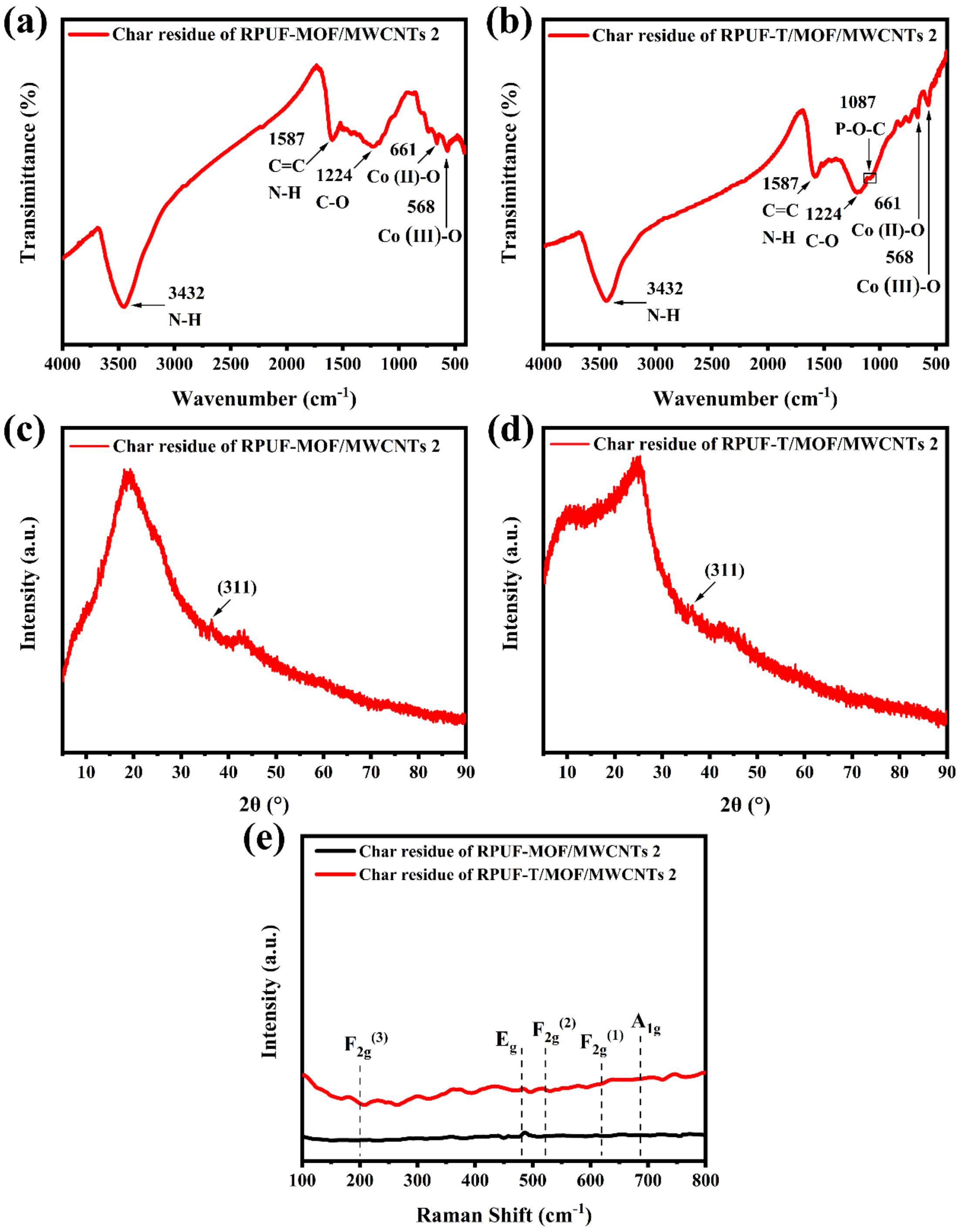

Figure S14. FT-IR spectrum (a), XRD curve (c) of char residue of RPUF-

MOF/MWCNTs 2, FT-IR spectrum (b), XRD curve (d) of char residue of RPUF-T/MOF/MWCNTs 2, Raman spectra (e) of char residues of RPUF-MOF/MWCNTs 2 and RPUF-T/MOF/MWCNTs 2.

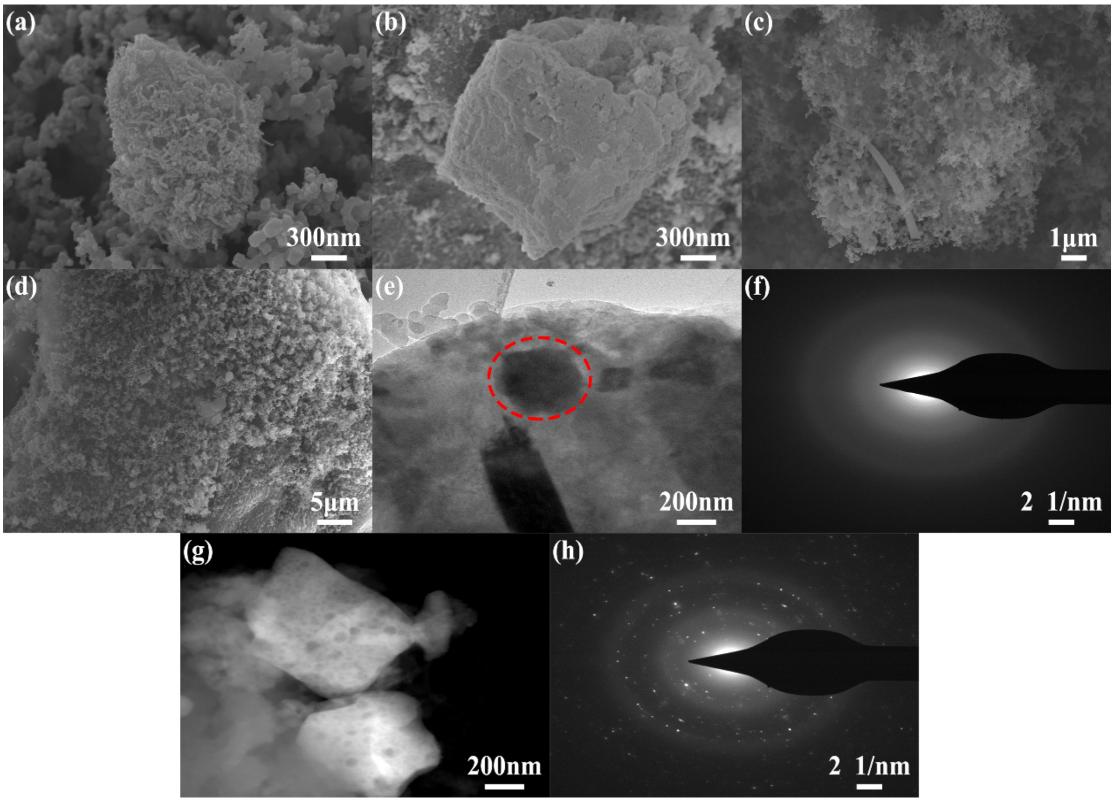

Figure S15. FSEM images (a, b), TEM image (e) and SAED pattern (f) of char residue of RPUF-MOF/MWCNTs 2, FSEM images (c, d), TEM image (g) and SAED pattern (h) of char residue of RPUF-T/MOF/MWCNTs 2.

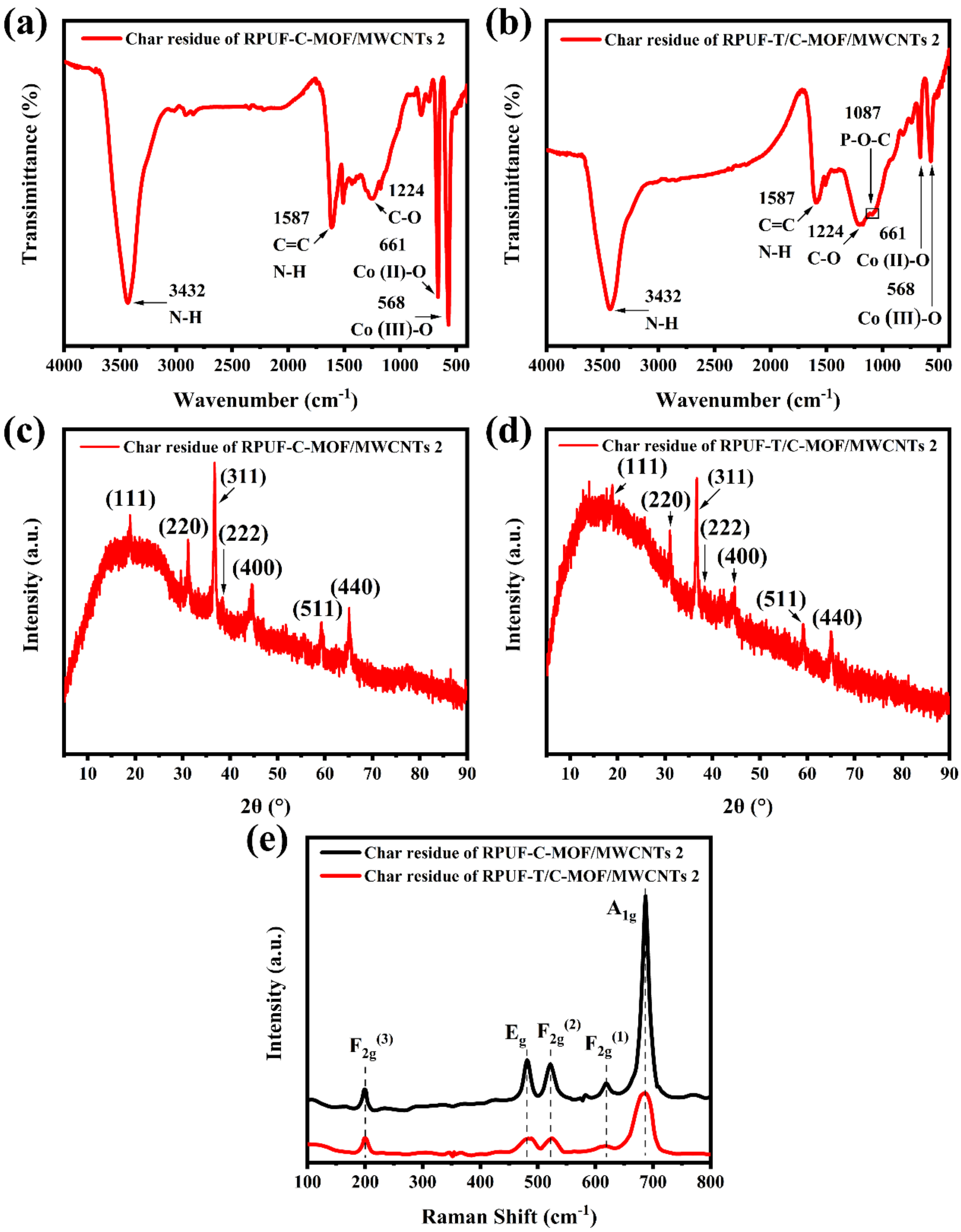

Figure S16. FT-IR spectrum (a), XRD curve (c) of char residue of RPUF-C-MOF/MWCNTs 2, FT-IR spectrum (b), XRD curve (d) of char residue of RPUF-T/C-MOF/MWCNTs 2, Raman spectra (e) of char residues of RPUF-C-MOF/MWCNTs 2 and RPUF-T/C-MOF/MWCNTs 2.

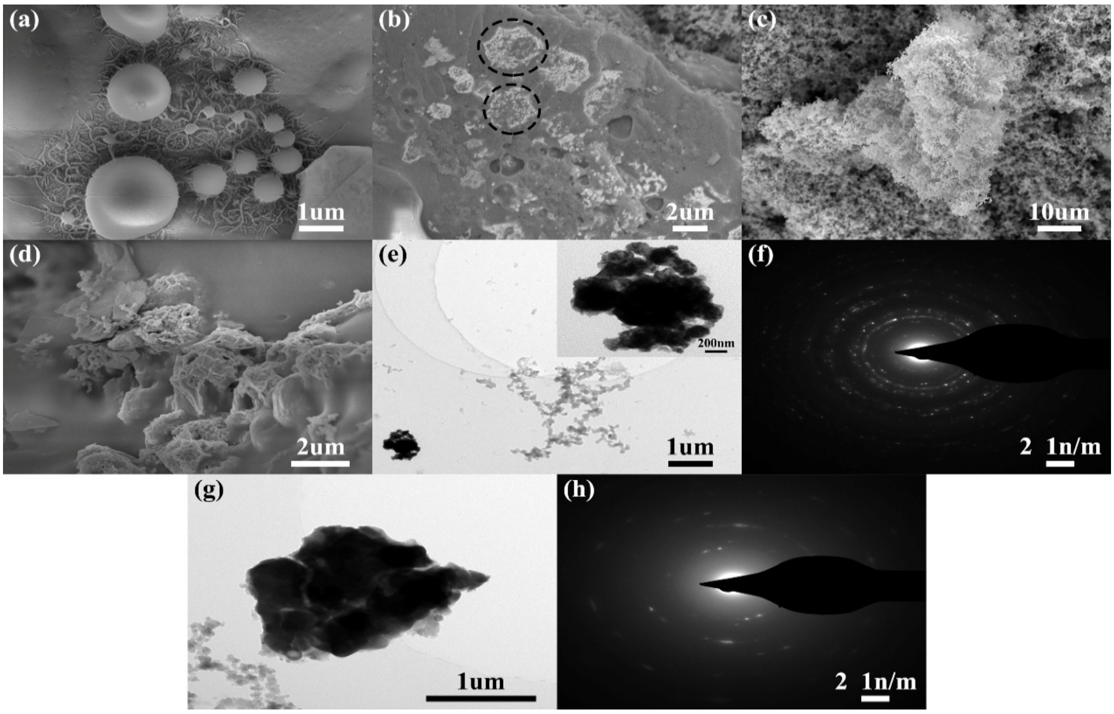

Figure S17. FSEM images (a, b), TEM image (e) and SAED pattern (f) of char residue of RPUF-C-MOF/MWCNTs 2, FSEM images (c, d), TEM image (g) and SAED pattern (h) of char residue of RPUF-T/C-MOF/MWCNTs 2.

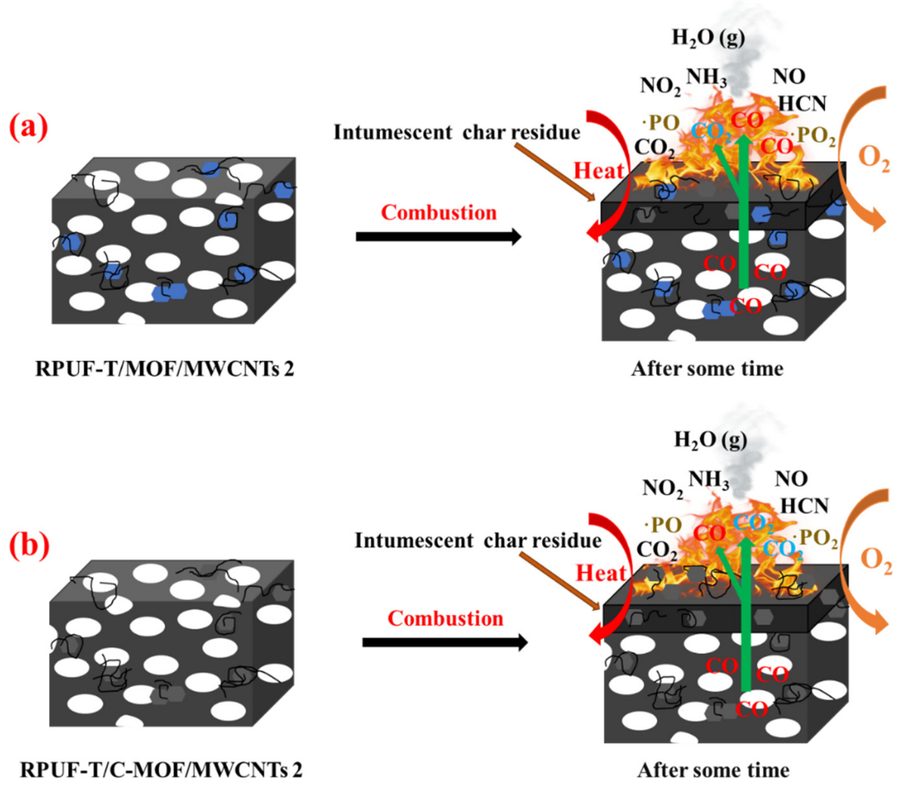

Scheme S1. Possible flame-retardant and catalytic CO mechanisms of RPUF-T/MOF/MWCNTs 2 (a) and RPUF-T/C-MOF/MWCNTs 2 (b).

Table S3. The proportion of elements of XPS curves of MOF/MWCNTs and C-MOF/MWCNTs.

Sample	Component	Atom (%)	Mass (wt%)
	C1s	79.3/70.4	66.7/50.0
	N1s	9.9/1.1	9.8/0.9
A/B	O1s	7.1/20.0	7.9/18.9
	Co2p	2.9/6.0	11.8/20.9
	Cu2p	0.8/2.4	3.7/9.2

Note: 1. Sample A refers to MOF/MWCNTs.

2. Sample B refers to C-MOF/MWCNTs.

Table S4. The mass ratio and atom ratio of elements of FSEM image of C-MOF/MWCNTs with mapping mode.

Element	Mass (wt%)	Atom (%)
C	42.86	68.52
N	0.00	0.00
O	14.74	17.69
Co	41.41	13.49
Cu	0.98	0.30

Table S5. The mass ratio and atom ratio of elements of TEM image of C-MOF with mapping mode.

Element	Mass (wt%)	Atom (%)
C	7.27	21.42
N	1.73	4.38
O	12.45	27.53
Co	67.31	40.41
Cu	11.24	6.26

Table S6. TGA and DTG data of RPUFs under N_2.

Samples	$T_{5\%}$	$T_{50\%}$	$Rmax_1/Tmax_1$	$Rmax_2/Tmax_2$	Residue
	°C	°C	%·min^{-1}/°C	%·min^{-1}/°C	wt%
neat RPUF	249.04	367.04	-7.03/307.04	-2.88/425.04	16.89
RPUF-MOF/MWCNTs 1	261.04	371.04	-7.01/307.04	-1.99/401.04	19.66
RPUF-MOF/MWCNTs 2	265.04	385.04	-5.95/311.04	-2.67/417.04	21.27
RPUF-MOF/MWCNTs 3	267.04	395.04	-5.84/313.04	-2.55/411.04	22.86
RPUF-T	199.04	375.04	-3.87/307.04	-3.27/379.04	24.79
RPUF-T/MOF/MWCNTs 2	205.04	379.04	-4.06/315.04	-2.86/373.04	25.79
RPUF-C-MOF/MWCNTs 2	263.04	387.04	-6.33/311.04	-2.73/415.04	21.57
RPUF-T/C-MOF/MWCNTs 2	201.04	375.04	-4.37/313.04	-2.85/375.04	27.39

Table S7. TGA and DTG data of RPUFs under air.

Samples	$T_{5\%}$	$T_{50\%}$	$Rmax_1/Tmax_1$	$Rmax_2/Tmax_2$	Residue
	°C	°C	%·min^{-1}/°C	%·min^{-1}/°C	wt%
neat RPUF	260.48	452.48	-7.29/294.48	-7.06/502.48	0.47
RPUF-MOF/MWCNTs 1	232.48	436.48	-5.28/314.48	-6.55/540.48	0.53
RPUF-MOF/MWCNTs 2	238.48	442.48	-4.85/314.48	-5.67/528.48	0.55
RPUF-MOF/MWCNTs 3	254.48	450.48	-4.09/314.48	-8.53/514.48	0.59
RPUF-T	194.48	450.48	-4.06/272.48	-6.21/480.48	6.49
RPUF-T/MOF/MWCNTs 2	198.48	466.48	-2.18/277.48	-5.39/486.48	6.71

RPUF-C-MOF/MWCNTs 2	272.48	466.48	-4.13/318.48	-8.69/520.48	3.24
RPUF-T/C-MOF/MWCNTs 2	232.48	518.48	-2.22/304.48	-5.70/548.48	7.42

Table S8. The proportion of elements of MOF/MWCNTs and B-MOF/MWCNTs.

Sample	Component	Atom (%)	Mass (wt%)
	C1s	79.3/76.2	66.7/57.6
	N1s	9.9/1.6	9.8/1.4
A/B	O1s	7.1/15.5	7.9/15.6
	Co2p	2.9/5.3	11.8/19.7
	Cu2p	0.8/1.4	3.7/5.7

References:

1. Wang, H.; Qiao, H.; Guo, J.; Sun, J.; Li, H.; Zhang, S.; Gu, X. Preparation of cobalt-based metal organic framework and its application as synergistic flame retardant in thermoplastic polyurethane (TPU). *Composites Part B: Engineering* **2020**, 182, 107498, doi:10.1016/j.compositesb.2019.107498.
2. Li, X.; Gao, X.; Ai, L.; Jiang, J. Mechanistic insight into the interaction and adsorption of Cr(VI) with zeolitic imidazolate framework-67 microcrystals from aqueous solution. *Chem. Eng. J.* **2015**, 274, 238-246, doi:10.1016/j.cej.2015.03.127.
3. Yao, J.; Chen, R.; Wang, K.; Wang, H. Direct synthesis of zeolitic imidazolate framework-8/chitosan composites in chitosan hydrogels. *Micropor. Mesopor. Mat.* **2013**, 165, 200-204, doi:10.1016/j.micromeso.2012.08.018.
4. Hu, Y.; Kazemian, H.; Rohani, S.; Huang, Y.; Song, Y. In situ high pressure study of ZIF-8 by FTIR spectroscopy. *Chem. Commun.* **2011**, 47, 12694, doi:10.1039/c1cc15525c.
5. Gross, A.F.; Sherman, E.; Vajo, J.J. Aqueous room temperature synthesis of cobalt and zinc sodalite zeolitic imidizolate frameworks. *Dalton T.* **2012**, 41, 5458, doi:10.1039/c2dt30174a.
6. Xu, W.; Wang, X.; Wu, Y.; Li, W.; Chen, C. Functionalized graphene with Co-ZIF adsorbed borate ions as an effective flame retardant and smoke suppression agent for epoxy resin. *J. Hazard. Mater.* **2019**, 363, 138-151,

doi:10.1016/j.jhazmat.2018.09.086.

7. Sadezky, A.; Muckenhuber, H.; Grothe, H.; Niessner, R.; Pöschl, U. Raman microspectroscopy of soot and related carbonaceous materials: Spectral analysis and structural information. *Carbon* **2005**, 43, 1731-1742, doi:10.1016/j.carbon.2005.02.018.
8. Li, M.; Wang, S.; Han, L.; Yuan, W.; Cheng, J.; Zhang, A.; Zhao, H.; Wang, Y. Hierarchically porous SiO2/polyurethane foam composites towards excellent thermal insulating, flame-retardant and smoke-suppressant performances. *J. Hazard. Mater.* **2019**, 375, 61-69, doi:10.1016/j.jhazmat.2019.04.065.
9. Tai, Q.; Hu, Y.; Yuen, R.K.K.; Song, L.; Lu, H. Synthesis, structure–property relationships of polyphosphoramides with high char residues. *Journal of materials chemistry* **2011**, 21, 6621, doi:10.1039/c0jm03959d.
10. Wang, P.; Xia, L.; Jian, R.; Ai, Y.; Zheng, X.; Chen, G.; Wang, J. Flame-retarding epoxy resin with an efficient P/N/S-containing flame retardant: Preparation, thermal stability, and flame retardance. *Polym. Degrad. Stabil.* **2018**, 149, 69-77, doi:10.1016/j.polymdegradstab.2018.01.026.
